# Supplementary material for: A systematic review of qualitative research on the physical and mental health impacts of immigration detention on asylum seekers and refugees
Source: PLOS Glob Public Health. 2025 Oct 29;5(10):e0005196. doi: 10.1371/journal.pgph.0005196 (PMC12571329; doi:10.1371/journal.pgph.0005196)
Supplement: S1 Table — (DOCX) [file pgph.0005196.s001.docx]

**Supplementary File 2: Quality Assessment Utilising the Joanna Briggs Institute (JBI) Checklist for Qualitative Research**

| *Study* | Q1. S there congruity between the stated philosophical perspective and the research methodology? | Q2. Is there congruity between the research methodology and the research question or objectives? | Q3. Is there congruity between the research methodology and the methods used to collect data? | Q4. Is there congruity between the research methodology and the representation and analysis of data? | Q5. Is there congruity between the research methodology and the interpretation of results? | Q6. Is there a statement locating the researcher culturally or theoretically? | Q7. Is the influence of the researcher on the research, and vice- versa, addressed? | Q8. Are participants, and their voices, adequately represented? | Q9. Is the research ethical according to current criteria or, for recent studies, and is there evidence of ethical approval by an appropriate body? | Q10. Do the conclusions drawn in the research report flow from the analysis, or interpretation, of the data? |
| --- | --- | --- | --- | --- | --- | --- | --- | --- | --- | --- |
| Arsenijevic et al. 2018 | UNCLEAR | YES | YES | YES | YES | NO | NO | YES | YES | YES |
| Arshad et al. 2018 | UNCLEAR | YES | YES | YES | YES | NO | NO | YES | YES | YES |
| Boerma et al, 2022 | UNCLEAR | YES | YES | YES | YES | NO | NO | YES | N/A | YES |
| Campbell et al. 2013 | YES | YES | YES | YES | YES | NO | NO | YES | YES | YES |
| Cleveland et al. 2018 | UNCLEAR | YES | YES | YES | YES | NO | NO | YES | YES | YES |
| Coffey et al., 2010 | UNCLEAR | YES | YES | YES | YES | NO | NO | YES | YES | YES |
| Diaz et al. 2023 | UNCLEAR | YES | YES | YES | YES | NO | NO | YES | YES | YES |
| Hollis, 2018 | YES | YES | YES | YES | YES | YES | NO | YES | YES | YES |
| Johnston et al., 2009 | YES | YES | YES | YES | YES | YES | NO | YES | YES | YES |
| Kellezi et al. 2016 | UNCLEAR | UNCLEAR | UNCLEAR | UNCLEAR | YES | NO | NO | YES | NO | YES |
| Kronick et al. 2015 | YES | YES | YES | YES | YES | NO | NO | YES | YES | YES |
| Kronick et al. 2018 | YES | YES | YES | YES | YES | NO | YES | YES | YES | YES |
| Mares & Jureidini, 2004 | UNCLEAR | YES | YES | UNCLEAR | YES | NO | NO | YES | YES | YES |
| Marquez et al. 2021 | YES | YES | YES | YES | YES | NO | NO | N/A | N/A | NO |
| Passardi et al. 2022 | YES | YES | YES | YES | YES | YES | NO | YES | YES | YES |
| Puthoopparambil et al. 2015 | UNCLEAR | YES | YES | YES | YES | NO | NO | YES | YES | YES |
| Rothe et al., 2003 | UNCLEAR | UNCLEAR | UNCLEAR | UNCLEAR | YES | NO | NO | YES | NO | YES |
| Shishehgar et al. 2021 | UNCLEAR | YES | YES | YES | YES | YES | YES | YES | YES | YES |
| Witney and Bates 2016 | YES | YES | YES | YES | YES | NO | NO | YES | NO | YES |
| Zimmerman et al., 2012 | YES | YES | YES | YES | YES | NO | No | Yes | Yes | Yes |
