# Supplementary material for: A systematic review of qualitative research on the physical and mental health impacts of immigration detention on asylum seekers and refugees
Source: PLOS Glob Public Health. 2025 Oct 29;5(10):e0005196. doi: 10.1371/journal.pgph.0005196 (PMC12571329; doi:10.1371/journal.pgph.0005196)
Supplement: S1 Data — (DOCX) [file pgph.0005196.s003.docx]

**Table 1 Characteristics and key findings of the impacts of immigration detention on ASR (n=20)**

| **Author and year** | **Phenomena of interest** | **Sample and participant characteristics** | **Country of origin** | **Country of study** | **Method for data collection and analysis** | **Description of main results** |
| --- | --- | --- | --- | --- | --- | --- |
| **Arsenijevic et al, (2018) [72]** | Self-perceived vulnerabilities of male migrants travelling alone along the Balkan route to Europe.  Detention duration not specified | 30 adult male migrants travelling along the Balkan route | Afghanistan, Pakistan, Algeria, Iraq, Syria, Morocco | Serbia | Individual interviews, group interviews and participant observation.  Data was analysed using thematic content analysis. | Participants describe the precarious living conditions in most of the detention camps, including the overall lack of food, extremely poor sanitary conditions, overcrowding and high incidence of diseases, and limited or no access to medical care. There was an instance of a detention camp where participants were reportedly kept in cages, in inhumane and undignified living conditions. Dire mental health conditions of co-detainees were also described. |
| **Arshad et al (2018) [66]** | Pregnant migrant women’s experiences of living in detention.  Detention duration: 53- 212 days | 4 migrant women who had been experienced some/all of their pregnancy while in detention; two volunteer health professionals | Central Europe, Sub–Saharan Africa and India | UK | Semi-structured interviews.  Data was thematically analysed using Braun and Clarke’s six-stage approach. | Pregnant migrant women in detention are noted to be a vulnerable group with poor underlying mental and physical health that is exacerbated by the detention process. Four key themes emerged: 1) challenges in accessing maternity care, 2) exacerbation of mental health conditions, 3) feeling hungry, and 4) lack of privacy. Results suggest that women face significant challenges in the access of timely and appropriate maternity care, their health concerns are disregarded and they experience a lack of continuity of care. |
| **Boerma et al., (2022) [73]** | Experiences of adult refugees who had been held in immigration detention in the Netherlands and the impact of this on their mental health.  Detention duration: Range 19 – 365 days. | 9 adult ASR (7 M, 2 F); age range 19-44 years) who were treated for post-traumatic stress disorder at a specialist mental health facility and who had previously been in immigration detention | Sub-Saharan Africa, Central Asia | Netherlands | Semi-structured individual interviews guided by a topic guide.  Data was analysed using grounded theory with codes being grouped and merged, later utilising axial coding. | All participants reported that their experiences in immigration detention were significantly impactful and detrimental to their mental health. The interviews revealed the following primary themes: uncertainty regarding expectations, the perception of being treated as criminals, anticipation of protection upon arrival in the Netherlands, and the experience of re-traumatization. These perceptions engendered feelings of anxiety, hopelessness, shame, anger, and suicidal tendencies. A significant number of participants were subjected to isolation, which exacerbated their mental health conditions. |
| **Campbell and Steel (2014) [12]** | Experiences of asylum seekers detained in Australia to explore the relationship between mental wellbeing, living conditions, and Australia’s detention policies.  Detention duration not specified | 7 adult asylum seekers (6 M, 1 F) who had previously lived in a range of Australian detention facilities;3 health and social-work professionals | Sri Lanka, Iran, Iraq, Afghanistan, Pakistan | Australia | Ethnographic observations, semi- structured interviews, key-informant interviews, and document analysis of international human rights protocols.  Data was analysed using Charmaz’s methods for constructivist grounded theory. | Results highlight the mental distress suffered by asylum seekers in detention, the environments of constraint in which they live, and aspects of detention centre policy that contribute to these environments. A discrepancy between asylum seekers’ experiences under immigration detention policy and Australia’s human rights obligations is also reported. |
| **Cleveland et al, (2018) [68]** | Adult asylum seekers’ experience of detention in Canadian immigration detention centers  Mean detention: 31 days | 81 adult asylum seekers held in two Canadian immigration detention centers at the time of the study. | Sub-Saharan Africa, South Asia, Middle East and North Africa, Latin America, Caribbean, Europe. | Canada | Semi-structured interviews.  Data was analysed using thematic content analysis. | Detention, even for brief periods in relatively adequate conditions, was found to be detrimental to asylum seekers’ mental health. This adverse impact appears to be largely attributable to the combined effect of two factors: symbolic violence and disempowerment. Asylum seekers express shock and humiliation at being ‘‘treated like criminals.’’ |
| **Coffey, (2010) [3]** | Experience of extended periods of immigration detention from the perspective of previously detained asylum seekers and consequences of these experiences for life after release  Mean detention: 3.17 years | 17 adult refugees (16 M, 1 F) who had been held in immigration detention funded by the Australian government. Participants were living in the community and had been granted permanent visa status or such status was imminent at the time of the study. | Afghanistan, Iran, Iraq, Other Middle-Eastern | Australia | Semi-structured interviews. (on average three years and eight months following participant’s release from detention). Data was analysed using thematic coding. | Participants struggle in rebuilding their lives in the years following release from immigration detention, with the majority experiencing pervasive difficulties. Participants endure an ongoing sense of insecurity and injustice, struggles with relationships, profound changes to view of self and poor mental health. Depression, demoralisation, concentration and memory disturbances, and persistent anxiety are commonly reported. |
| **Diaz et al, (2023) [2]** | Impact of the US immigration detention system on the health of people recently released by ICE.  Median detention: 11 months | 16 adult migrants (13M, 2 F, 1 non-binary) released from US immigration detention within two years of study enrolment | Dominican Republic, Jamaica, Trinidad and Tobago, Honduras, Mexico, Guatemala, Pakistan, Russia, Kazakhstan | USA | Semi-structured anonymous phone interviews. Data was processed using reflective thematic analysis with an inductive approach. | Four themes emerge from analysis: 1) poor conditions and inhumane treatment, 2) a pervasive sense of injustice, 3) structural barriers limiting access to care, and 4) negative health impacts of immigration detention. |
| **Hollis, (2018) [64]** | Explore the psychosocial stressors experienced by people in detention, the psychological impacts of being detained and the ways in which people express resilience and cope in detention.  Mean duration: 3 months (Range 24 hours- 7 months) | 9 adult asylum seekers (7 M, 2 F; mean age 31) who had previously been held in UK IRCs. | Iran, Iraqi- Kurdistan, Pakistan, Yemen, Zimbabwe | UK | Semi-structured interviews conducted guided by a topic guide.  Data was analysed using interpretive phenomenological analysis. | Participants exhibited incredulity and cognitive dissonance regarding their detention, resulting in their deprivation of communication and healthcare. These stressors culminated in feelings of powerlessness, self-doubt, and contemplation of their uncertain futures. Nevertheless, participants demonstrated resilience by adopting proactive behaviours, engaging in spiritual practices, and relying on personal relationships to cope during their time in detention. |
| **Johnston, et al (2009)[74]** | Association of Australian asylum policies and practices with adverse health outcomes  Mean detention duration <12 months | 60 adult refugees with Temporary Protection Visas (TPV) living in the community, with a previous experience of immigration detention | Iraq | Australia | Semi-structured interviews.  Data was analysed using thematic coding. | Participants generally feel socially isolated and lacking in control over their life circumstances, because of their experiences in detention and on a temporary visa. Perception of injustice in immigration detention is a significant cause of anger, with many feeling that that detention policies violate their human rights. The disrespectful treatment of women, lack of access to female doctors for Muslim women, and the overall loss of freedom are highlighted as central concerns in immigration detention. |
| **Kellezi & Bosworth, (2016)[67]** | Mental health, self-harm and suicide in detention (UK immigration removal centers, IRC).  Detention duration not specified | 19 adult migrant women detained in an IRC at the time of the study; 6 members of staff | Not reported | UK | Semi-structured interviews.  Details of data analysis not reported. | Most detainees report very high levels of anxiety, distress and depression. Nights, early days in detention, negative immigration outcomes, concerns about medical and physical health, witnessing other detainees in distress or being removed, and separation from family (especially children) are described as particularly difficult. |
| **Kronick et al, (2015) [76]** | Experiences of detained children and families who have sought asylum in Canada.  Mean detention duration 56.4 days | 20 families (65% asylum seekers, 35% failed refugee claimants) comprising of 35 children in total. 12 families were interviewed during detention and 8 families post release from detention. | Europe, Southwest Asia, West Africa, East Africa, North America, Middle east, Central Africa, South Asia, Latin America | Canada | Semi-structured interviews and ethnographic participant observation.  Data was analyzed through inductive coding guided by thematic analysis principles.  Inizio modulo  Fine modulo | Detention is described as a frightening experience of deprivation leaving children feeling criminalized and helpless. Family separation is noted to further shatter children’s sense of well-being. Children’s emotional and behavioural responses to separation and to detention indicates experiences of acute stress and, in some cases, trauma. Even when detention is brief, distress and impairment often persisted months after release. |
| **Kronick et al, (2018) [70]** | Lived experiences of children held in Canadian immigration detention.  Mean detention duration: 90.4 days | 10 children from 8 different families, either living in detention or with an experience of immigration detention at the time of the study. (Mean age: 8.5 years; range 3-13) | Not reported | Canada | Sand-play and narrative inquiry – data included photos and drawings of sand-trays, interviews with parents and key informants, transcriptions of sand-play stories, field notes. Data was analysed using coding themes for sand-trays and sand-play stories, and re-contextualized in data gathered from field notes and parents’ interviews. | The interpretation of children’s oral narratives and symbolic worlds generates three broad themes: 1) confinement and surveillance; 2) loss of protection; and 3) human violence. |
| **Mares & Jureidini, (2004) [75]** | Mental health (of children and families living in immigration detention  Mean detention duration: 1.25 years (range 12-18 mo) | 16 adults (7M, 9 F) and 20 children from 10 families who, at the time of the study, were being detained in a remote Immigration and Reprocessing Centre (IRPC) and had been referred to a specialist mental health service. | Iran, Iraq, Afghanistan, Palestine | Australia | Multiple, detailed clinical interviews, undertaken by a range of experienced mental health clinicians at a specialist mental health service,  Details of data analysis not reported. | There are high levels of psychiatric distress among both adults and children. All children had at least one parent with psychiatric illness, with the majority of adults meeting criteria for MDD and PTSD. Children under five showed developmental delays and attachment disturbances; all had been exposed to violence and chronic parental mental illness. Older children exhibit PTSD symptoms related to traumatic experiences in detention, including witnessing severe self-harm and suicidal behaviour. They struggled with sleep, concentration, and feelings of hopelessness, and reported anxiety about parental well-being. Parental incapacity due to mental illness often left children assuming caregiving roles, exacerbating their emotional burden. |
| **Marquez et al (2021)[21]** | Policies and prevention measures for detainee suicides within Immigration Customs Enforcement (ICE) detention facilities  Detention duration not specified | N/A | Latina American, other (not reported) | USA | Inspection reports detainee death reports , and intergovernmental contracts from 116 large ICE detention facilities  Data was analysed using thematic coding with an inductive approach. | Detention facilities were noted to be inadequately staffed and resourced, resulting in the failure to implement federally mandated protocols regarding detainees’ well-being competently and promptly. |
| **Passardi et al, (2022)[15]** | Moral injury appraisals and associated mental health outcomes related to immigration detention on Nauru.  Detention duration: 14.2 months (SD = 11.1) | 13 adults who had sought (9F, 4M) refuge in Australia and, due to arriving by boat, had been transferred to immigration detention on Nauru. Participants were living in Australia for 28 to 64 months following their medical evacuation (M= 55.3, SD= 12.3) and had spent between 1 and 36 months in Nauru (M= 14.2, SD= 11.1). | Iran, Somalia, Nepal, Sri Lanka, others | Australia | Semi-structured in-depth interviews.  Data was analysed using reflexive thematic analysis. | Participants met the criteria for PTSD (n=10), Major depressive disorder (n=9), panic disorder (n=3), and obsessive-compulsive disorder (n=3); only two had no current disorder(s). Four participants expressed suicidal ideation, three had past suicide attempts more than two years prior, and one participant had made a suicide attempt less than two years prior. Major themes included participant experience of deprivation, lack of agency, violence, and dehumanisation after arrival, with the Australian government seen as the driving force behind these experiences. These experiences culminate in feeling irreparably damaged for ASR with an experience of detention. |
| **Puthoopparambil et al, (2015) [13]** | Perceptions and experiences of immigrant detainees in Swedish immigration detention centres.  Detention duration: 2 weeks – 9 months | 21 adult immigrants (19 M; 3 F) detained in three Swedish immigration detention centres at the time of the study. | Belarus, Chile, Guinea, Iraq, Morocco, Nicaragua, Palestine, Senegal, Serbia, Sierra Leone, Uganda and the USA | Sweden | Semi-structured interviews  Interview transcripts were analysed using thematic analysis (Braun and Clarke, 2006) | The detainees liken immigration detention to imprisonment. There is a lack of control over their life mainly due to arbitrary restrictions and inadequate response from authorities, which make seeking help appear futile. This perceived lack of control forced them into passivity. Differences in amenities provided in different centres were observed and some of these were reported to help in making detention more bearable. |
| **Rothe, (2003) [71]** | Psychopathology of refugee adolescents confined to detention in Guantanamo Bay.  Detention duration: 6-8 months | 74 adolescents (47 M, 27 Female; mean age 15.5 years) who had left Cuba by sea en route to the USA but who were intercepted and confined to detention in Guantanamo Bay, where they were held at the time of the study and referred to a psychiatric clinic for evaluation. 66% of the boys and 70% of the girls were accompanied by both parents. | Cuba – ethnicity not specified | USA (Guantanamo Bay) | Clinical interviews, including projective drawings  Details on data analysis not reported. | Notable themes developed included: evidence for pre-existing vulnerability to trauma; presence of post-traumatic and regressive symptoms and behavioural problems; interaction of these symptoms with lives in the camp; experiences of dehumanization inside the camps. |
| **Shishehgar et al, (2021) [14]** | Experiences of asylum seeker women living in Australian immigration detention and impact on mental health.  Detention duration: 1-4 months (range) | 17 adult Iranian female asylum seekers, previously detained and living in the Australian community for 2-3 years on bridging visas at the time of the study. | Iran | Australia | Semi-structured interviews  Data was analysed using thematic and inductive analysis. | Living in a prison-like environment was described as a punishment for their boat arrival, violating their privacy and dignity. In contrast, a sense of security, free access to healthcare services, and the possibility to build social networks were positive aspects of short-term detention. |
| **Witney & Bates, (2016) [63]** | Experiences of immigrants previously held in Australian immigration detention and the incorporation of their traumatic experiences within the self-construct  Detention duration >2 years | 10 adult male former asylum seekers living in the community on TPV and permanent protection visas (PPV) after being held in mandatory detention ; 10 males age 19-51. | Not reported | Australia | Semi-structured interviews.  Data was analysed using thematic analysis. | Before receiving their visas, all participants had been held in detention centres onshore on the Australian mainland (n=7) or offshore in neighbouring Pacific islands (n=3) for periods ranging from 3 to 7 years. Interviews occurred around six months post-release from detention into the community. Thematic analysis revealed that participants experienced similar levels of narrative disturbances as a result of detention, which affected their sense of past, present, and future. However, their subsequent narrative integration varied. Three groups representing different levels of narrative integration were identified: robust integration, limited integration, and fragmentation. Higher levels of integration were linked to a greater sense of unity and purpose in their stories. |
| **Zimmerman et al., (2012) [65]** | Exploratory study seeking to test the usefulness of a questionnaire among former immigration detainees examining perceptions of their health needs and access to care within immigration detention in the UK.  Detention duration: median 185 (IQR: 10-990) day | 30 adult participants (26 M, 4 F; mean age 33) of whom 27 were asylum seekers and 3 were detained on judicial grounds (without seeking asylum thus excluded). All had been released from detention within the past 12 months. | Afghanistan, Iran, Nigeria, Somalia, Morocco, Zimbabwe, Palestine, Benin, Ghana | UK | Semi-structured interviews guided by a interview topic guide.  Data was analysed using thematic analysis. | The findings indicate that 66 percent of participants entered detention with pre-existing mental or physical health conditions necessitating ongoing or new treatments. Furthermore, the majority of participants experienced the emergence of new mental/emotional (93 percent) or physical (53 percent) health issues during their detention. Access to healthcare within the detention environment was frequently hampered by various formal and informal challenges, affecting both its effectiveness and availability. The authors conclude that this exploratory study raises significant concerns regarding the adequacy of mental and physical health care provisions for detainees within the current UK detention system. |

*Note.* ICE = Immigration Customs Enforcement; IRC = Immigration Removal Centres; PPV = Permanent Protection Visa; TPV = Temporary Protection Visa
