# Supplementary material for: A systematic review of qualitative research on the physical and mental health impacts of immigration detention on asylum seekers and refugees
Source: PLOS Glob Public Health. 2025 Oct 29;5(10):e0005196. doi: 10.1371/journal.pgph.0005196 (PMC12571329; doi:10.1371/journal.pgph.0005196)
Supplement: S2 Data — (DOCX) [file pgph.0005196.s004.docx]

**Table 2 Selected Supporting Quotes from Studies**

| Themes and sub-themes | Supporting quotes | Contributing studies |
| --- | --- | --- |
| Detention Period | | |
| Human Rights Violations in Detention   - Constriction of agency and autonomy - Endemic uncertainty - Systemic Deprivation - Dehumanisation and perceived injustice - Loss of safety | - “.. just being isolated, no privacy, men walking in ... feeling powerless….I couldn’t stop crying. I didn’t expect this, after what I had just been through in my country.’’ - “It is like you are a big criminal, you are there even though you never did any crime, or you never did anything wrong…but they are watching you.,,every step you take from outside your room…. If they make all the walls or fence with gold, there is nothing different, there is nothing changed, prison is prison. Still this system keeps me in detention for no reason.” - “There is no justice in detention in the eyes of the detainee; some people are released very quickly, some may stay there like I did for more than two years, and some may be there for five years.” - "I didn’t even know [where I was]. Because they didn’t even tell me. They just brought a letter for me. And it was in Dutch also. I have to sign this letter. Okay." - “you’re locked up in a box... you don’t even get sunlight. You’re begging for sunlight, imagine that.” - "I didn’t do nothing and they handcuffed me and [...] when I reached there, I want to ask like: 'I want to pee first' and there it was a police eh..., with me in the toilet. I don’t know the reason why." - “The first night in detention I can never forget. When I was put in a single cell with no food. And I was hungry and lonely. Because this happened in a country where there should be human rights and respect for human rights. I can never forget that.” - “This is such an unfair policy, especially for children. They come here looking for a safe place; they are not criminals. We are just normal people looking for a safe place...There are no human rights coming to us here...We are all people looking just for freedom but when they put us in this jail, they take the most important thing for us; our freedom.” - “It’s like a prison... My dad was living in another building. He was separated from us.... Here, this guy was watching us so we could not go outside... These guys were watching us not to flee” - “‘Life is like ... we are like garbage. A dog is better than a man… they put 30 of you in a room like cows, so how can you sleep?” - “We were kept in a prison, given a room in the camp. It looked like a cage ... Officers entered occasionally into my room without knocking the door ... I wanted to go back to Iran from the Christmas Island detention.” - “They took us to another camp, made of fences, like cages. They put 40–50 men in closed fenced cages. It was like a really big hall, closed, and inside that, there were fences. They gave us some food in the morning and evening, a really small piece, just so that you can stay alive with it. Just once in 24 hours you were allowed to go to the toilet and there were no facilities to take a shower... The fences are locked. You are not able to go out. The living conditions were really bad, really terrible..” - “The red light in the room at night, its . . . to see if all detainees are in the room . . . I mean, they follow you like . . . like criminals. So, they do not speak to you, they watch you.” - “We feel as an animal. It’s not what they do to humans. That time, I felt they were looking at me as an animal. But when they transferred me to Australia, I thought, no, I thought wrong because the people have a very nice behaviour with pets. When I went to the shop for the first time, I saw lots of food for dogs or cats. They have got toilets, they have got doctors. They have everything they need. So, we are smaller than that for the people who work in the detention camp or government.” - “One behaviour from the officers that we didn’t really like and we actually wrote complaints about that. We found it very assaulting and insulting when they used to stand behind the fences and throw bags of clothing to us without even coming over as if we’re ill or sick people or have got something contagious . . . You know, all of us, my friends, my family and I, were so upset about that. We were saying “Do we have a skin condition that you just don’t want to be in contact?”’ | Arsenijevic, 2018  Arshad, 2018  Boerma, 2022  Cleveland, 2018  Coffey, 2010  Diaz, 2023  Hollis, 2018  Johnston, 2009  Kronick, 2015  Kronick, 2018  Passardi, 2022  Puthoopparambil, 2015  Shishehgar, 2021 |
| Systemic Failures of Healthcare Provision and resultant ASR vulnerability and deprivation   - Institutional and organisational failure - Structural barriers to medical care | - “I would like to see the midwife because I would like to know what’s happening … I just want to hear my baby heart beat ... so I would wait all day but the midwife never come back to see me.” - “Before I saw the doctor – he did not help me for the pain of my tooth. And the security guard helped me and I took one of my teeth out myself.” - “I’d write on the screen, I need to talk to the psychologist, please, it’s urgent, I feel depressed, please help me... when you write sometime like that it’s because you really have a serious problem... 2-3 weeks later they’d say, okay, come over here.” - “In fact, the doctor told me pretty much, “’you’re not dead yet, so there’s no reason for us to help you yet.” - “We went to the labour ward ... the midwife ask the guards to leave because she need to check me ... the guard ask her to “leave the door open because we need to know what’s happening.” - “Because I’m not criminal. (...) I was shamed to go to hospital because people are thinking ‘Who is he, has he killed somebody here? (...) You know, when I come to this country, I come for the freedom. I was shamed to go to hospital with [shackles], I refuse them.” - “They disinfected everything, they wore gloves. Instead of handing me my food tray, they threw it on the table, as if I was an animal. (...) They didn’t respect me. I asked for help and that’s how they treated me.” - When I saw myself in the mirror, it gave me a very bad feeling about myself – about my health, my back. And no one helped. I was so sad because of this. - “I asked the nurses – I said I want to see the GP several times and they kept saying ‘‘we’ll call you’’. For almost 2 and a half months. There was no way. Just paracetamol is the only painkiller. You had to realize it was enough if you couldn’t get anything else.” - After release from detention doctors outside ‘‘started listening. The ones there weren’t listening. Now they ask about your previous life and what makes you upset – they treat you like a person. In detention they just give tablets’’ | Arsenijevic, 2018  Arshad, 2018  Campbell, 2014  Diaz, 2023  Hollis, 2018  Zimmerman, 2012 |
| Negative health impacts of immigration detention   - Psychological health - Physical health - Impact on children | - “As you can see, I have very high blood pressure. Everything is affecting me badly. Here we are stuck between life and death. Our life is ahead of us. Future, to go out ... to go home, go back and die. ... I am full of regret. Full of regret for my whole life. I wish I had never left. I came here and there’s nothing for us, there is no one to help us.” - "It was like, you know, sometimes if you have a wound. And someone is still peeling it again. [...] I have a wound. And they’re still adding more pain on me, again." - "Because in my mind, I was asking myself: what, what’s going to happen to me? [...] You know, sometimes in Africa, they kill people. Like how they kill them, is like cutting the part. They cut your hand. They cut your arm. Cut your finger. They cut you. They kill you in pain before you die." - “Most of the time, I just took the tablets, and I was unconscious on my bed, sleeping. I rarely ate lunch. [...] When I saw myself in the mirror, it gave me a very bad feeling about myself – about my health, my back. And no one helped. I was so sad because of this.” - “I had very severe morning sickness. Very severe, you cant imagine. I couldn’t go for six months in dining room. I never eat food, for six months of pregnancy. It was, just, orange in a whole day, one orange. Sometimes nothing. I cried for plain rice. Can I eat? But, I couldn’t, I couldn’t. They never allow the food in the room. [...] They just say no, it’s rule. They couldn’t give you food in the room.“ - “It’s the surroundings. It’s what you’re in. You are in a place with 250 other people – and of the 250, there’s 10 happy ones. And the rest are just really at various stages. From really desperate, to suicidal, to depressed. It’s not a place to be for any length of time.” - “Because the situation was so, so hard, that the Iranian boy – he hanged himself by rope. To kill himself. But he wasn’t successful. The situation then was so, so horrible. - “If I think of the places in detention centres where I was, I feel very upset and scared because I was not happy at all in there. [...] if I think of it I have the feel of shivering. And also, when I was in the detention I thought will I do something to harm myself, that how much I was upset and stressed out” - “If you were very strong, a strong, strong human, they put you in isolation.one day, two days, one month, two months. This makes you damaged. This makes you crazy. You just go into the blanket, and lower, and lower. And you are thinking under the blanket.” - “The worst thing was that day by day there was no answer; then there was no hope… For each rejection, I used to [go] into my room for one week. [I] couldn’t eat and [I had] a really strong sense of hopelessness. I was so tired, so scattered.” - “In my country they torture your body but in Australia they kill your mind” - “Living in detention, it’s like looking at oneself in a mirror. When I saw someone try to commit suicide; it’s exactly like I’m doing that to myself.” - “..for 4 days I couldn’t eat. I cried every day from morning to night. Yeah, I think he [my infant] became sick because he was away from his mother. He cried every day [too].” - “‘N’, ‘aged 12, drank coffee in an attempt to remain awake all night for fear that his depressed mother or psychotic father (whom he had witnessed dancing naked in the camp) might come to grief without his vigilance. He had been victimised by other detainees and guards because of his father’s bizarre and provocative behaviour.” - “We [siblings] said we are together, but they [detention officers] told us ‘here is Australia, sisters and brothers are not part of family. You and your husband and your child are a family’ ... My sister was detained in the single detention ... I am very dependent on my sister. It was important for me to be with my sister.” - “You know you can’t throw [away] your own background...the greatest torture of all is not being able to contact your family members and not being able to tell them what is happening to you.” | Boerma, 2022  Campbell, 2014  Coffey, 2010  Hollis, 2018  Mares, 2004  Passardi, 2022  Shishehgar, 2021  Witney, 2016 |
| Post-detention period | | |
| Changes in Self-construct, relationships and worldview   - Fragmentation of identity and changes in self-perception - Erosion of trust and social connectivity. | - “Detention has the effect that it makes you a person who becomes passive, lazy . [It] pacifies a person, their personality, and stops people from actively pursuing their goals.” - “As a normal human being I don’t have the courage to talk to people, normally. After four, five minutes my entire body will be shaking or shivery.” - “At the moment my relationship with [my family] is fine, but they don’t know what’s in me. I won’t tell them because they have suffered enough so there is no point to let them share my pain. Secondly, I want them to go on with their future, and I want them to be far from these issues.” - “I did not attend [English classes] because I believed that we will not be released from the detention centre and I thought ‘What for?’” - “I’m constantly aware of my actions because I’m afraid that if I do any slight thing wrong they might tell me that “you need to go back to detention”.’ | Coffey, 2010  Kronick, 2015  Witney, 2916 |
| Enduring impact on mental and physical health   - Continued psychological sequalae - Physical health deterioration. | - “But how is it possible one person should be in detention [for] five years? I missed my life. My children didn’t have a father, and my wife didn’t have a husband. It’s just not right.” - “I worry about my future. I am now 39 years old. The train I might have caught has left without me on board, and now it is too late to catch it. It is the train that leads to the destination of marriage and a family. I will be too old to be a father in the future.” - “Those kind of traumas don’t go away overnight... I am walking down the street and I am afraid that immigration could come at any moment to get me... I’ve just leveled off a little bit mentally, and I’ve stopped having those dreams of imprisonment, those nightmares. But you keep thinking that immigration may come any time. You don’t feel free.” - “Now I don’t know, who am I? I lost myself. Because I’m not that person I was, that person who came to Australia. I was healthy. I was active. I had a hard time but I was happy. But now I can’t laugh, I can’t cry, I can’t work. I can’t study. I’m living in very dark place. (P02f) - “I’m definitely not the person who walked into that place...I walked into that centre very healthy and I came out with four of my lower discs bulged. I’m left with permanent mental and physical disability.” - “We don’t feel [we can] completely cope with this society because of the past. [In detention] they hurt us, they humiliated us, and they punished us. They made us very small and worse than anyone. All this sad feeling [from the past] shows us anyone could be an enemy; anyone could hurt you in the same way. We lived in fear [in detention], and still it is same thing, still I feel the same thing.“ | Coffey, 2010  Diaz, 2023  Witney, 2016 |
