# Supplementary material for: A systematic review of qualitative research on the physical and mental health impacts of immigration detention on asylum seekers and refugees
Source: PLOS Glob Public Health. 2025 Oct 29;5(10):e0005196. doi: 10.1371/journal.pgph.0005196 (PMC12571329; doi:10.1371/journal.pgph.0005196)
Supplement: Attachment — Submitted filename: PGPH-D-25-00362_comments.pdf [file pgph.0005196.s007.pdf]

PLOS Global Public Health

A systematic review of qualitative research on the physical and mental health impacts of immigration detention on asylum seekers and refugees

--Manuscript Draft--

**Manuscript Number:**	PGPH-D-25-00362
**Article Type:**	Research Article
**Full Title:**	A systematic review of qualitative research on the physical and mental health impacts of immigration detention on asylum seekers and refugees
**Short Title:**	Qualitative Systematic Review of the Health of ASR with an experience of detention.
**Corresponding Author:**	Bafreen Sherif, BPharm, PgDipClinPharm, MSc<br>Monash University<br>Melbourne, AUSTRALIA
**Order of Authors:**	Bafreen Sherif, BPharm, PgDipClinPharm, MSc
	Debbie C. Hocking
	Susan Rees
	Letizia M. Affaticati
	Suresh Sundram
**Keywords:**	Refugees; asylum seekers; immigration detention; physical health; mental health; qualitative research
**Abstract:**	Background<br>Globally, the population of refugees and asylum seekers has reached unprecedented levels, prompting several host countries to implement immigration detention as a strategy to mitigate the influx of unauthorised arrivals. Previous research focusing on asylum seekers and refugees has predominantly utilised quantitative methods to assess the effects of immigration detention on mental and physical health outcomes. This study represents the first qualitative systematic review aimed at evaluating and synthesising qualitative research concerning the impact of immigration detention on the physical and mental well-being of adult and child asylum seekers and refugees (ASR) residing in middle- to high-income host countries.<br>Methods and findings<br>Between December 2021 and October 2024, across medical, allied health, and social sciences databases and grey literature (Embase, MEDLINE, PsychINFO, CINAHL Plus, Global Health, APA PsycARTICLES, Social Policy and Practice, Cochrane Library, Scopus, Open Grey).were systematically searched to identify original peer-reviewed articles describing qualitative findings relating to the experience of immigration detention among ASR in middle—to high-income countries. The included studies' references were also perused. The systematic review was registered with PROSPERO (CRD) 42022328867. Study selection, critical appraisal and data extraction were performed employing the Joanna Briggs Institute Critical Appraisal Checklist for Qualitative Research. Two reviewers identified relevant abstracts and articles. Synthesis of the included studies identified prominent themes relating to the effects of immigration detention on health outcomes in ASR adults and children. A total of 2,512 records were initially identified and abstracts reviewed, of which 564 full-text articles were assessed for eligibility. Twenty were included in the final review. The duration of detention ranged from 2 weeks to 38 months. The pooled studies included 374 adult asylum seekers and refugees and 139 children with a current or past history of immigration detention. A total of five major themes influencing the physical and mental health impacts of immigration detention emerged from the synthesis. Three were relevant to the detention period: i) Human rights violations in detention, ii) Systemic failures of healthcare provision and resultant ASR vulnerability and deprivation, and iii) Negative health impacts of immigration detention. Two themes were relevant to the post-detention period: iv) changes in self-construct, relationships and worldview, and v) enduring impact on mental and physical health.<br>Conclusions<br>This comprehensive review highlights the uniformly negative and persistent outcomes

Powered by Editorial Manager® and ProduXion Manager® from Aries Systems Corporation

	associated with immigration detention on the health of ASR adults and children. Findings may usefully inform future health and human rights considerations of migration policy.
Opposed Reviewers:	
Additional Information:	
Question	Response
**Financial Disclosure**<br><br>Enter a financial disclosure statement that describes the sources of funding for the work included in this submission and the role the funder(s) played. This includes grants and any commercial funding of the work or authors.<br><br>This statement will be typeset if the manuscript is accepted for publication.<br><br>*Please review the submission guidelines and the instructions link below for detailed requirements and guidance.*	The funders had no role in study design, data collection and analysis, decision to publish, or preparation of the manuscript.
**Competing Interests**<br><br>On behalf of all authors, disclose any competing interests that could be perceived to bias this work.<br><br>This statement will be typeset if the manuscript is accepted for publication.<br><br>*Please review the instructions link below and PLOS Global Public Health's competing interests policy to determine what information must be disclosed at submission.*	The authors have declared that no competing interests exist.
**Data Availability**<br><br>Before publication, Authors are required to make fully available and without restriction all data underlying their findings. Please see our PLOS Data	Data supporting this study are included within the article and/or supporting materials

Powered by Editorial Manager® and ProduXion Manager® from Aries Systems Corporation

Policy page for detailed information on this policy.<br><br>A **Data Availability Statement**, detailing where the data can be accessed, is required at first submission. Insert your Data Availability Statement in the box below.<br><br>Please see the data reporting section of our submission guidelines for instructions on what you need to include in your Data Availability Statement.<br><br>This statement will be typeset if the manuscript is accepted for publication.<br><br>*PLOS allows rare exemptions to address legal and ethical concerns. If you have legal or ethical restrictions, please use the box below to detail these in full sentences for the Journal team to consider.*	

9 February 2025

Attention: PLOS Global Public Health Editorial Board

Bafreen Sherif
Monash University, Dept of Psychiatry, School of Clinical Sciences, Faculty of Medicine, Nursing and Health Sciences, Monash University, Victoria, Australia

Dear Professors Kyobutngi, Pai and colleagues,

We wish to submit an original systematic review manuscript entitled "*A systematic review of qualitative research on the physical and mental health impacts of immigration detention on asylum seekers and refugees"* for consideration by PLOS Global Public Health.

We confirm that this work is original, has not been published elsewhere, and is not currently under consideration for publication elsewhere.

In this paper, we report a systematic review that evaluates and synthesises qualitative research concerning the impact of immigration detention on the physical and mental well-being of adult and child asylum seekers and refugees (ASR) residing in middle- to high-income host countries. This is significant because, to our knowledge, there has been no prior systematic review of the qualitative literature addressing this domain. This is particularly timely and globally relevant, given ongoing humanitarian crises have led to an unprecedented rise in the global population of refugees and asylum seekers. In response, several host countries have adopted immigration detention as a strategy to manage the influx of unauthorised arrivals.

This manuscript is appropriate for publication in PLOS Global Public Health, as it aligns with the journal's mission of disseminating research that investigates deeply rooted global inequities in public health, especially among underrepresented and historically marginalised

communities, with the aim of addressing the most pressing health challenges and disparities confronting our society today. This systematic review seeks to advance both clinical practice and health policy to explore the health of populations from a global perspective, transcending the boundaries of individual nations.

PLOS Global Public Health is the ideal platform for our work. We are confident that our findings will contribute significantly to understanding global health challenges and will resonate with the journal's readership.

We look forward to the opportunity to share our research with your esteemed journal and are eager to receive any guidance or submission requirements.

We have no conflicts of interest to disclose.

Please address all correspondence concerning this manuscript to me at bafreen.sherif@monash.edu or to Professor Suresh Sundram at suresh.sundram@monash.edu.

We appreciate your consideration of this manuscript.

Yours sincerely,

Bafreen Sherif

B.Pharm (Hons), PgDipClinPharm (Distinc), MScClinPharm (Hons), PhD Candidate, RegPharm AU/NZ

Department of Psychiatry
Faculty of Medicine, Nursing and Health Sciences, School of Clinical Sciences at Monash Health
Monash Medical Centre, Level 3, P Block, 246 Clayton Road, Clayton VIC 3168 Australia
T: +61 3 9594 1479 F: +61 3 9594 6499
E: suresh.sundram@monash.edu
www.monash.edu
ABN 12 377 614 012 CRICOS Provider 00008C

Manuscript

Click here to access/download;Manuscript;Manuscript 04_02_24_PLOSGlobalPubHealth.docx

A systematic review of qualitative research on the physical and mental health impacts of immigration detention on asylum seekers and refugees

Bafreen Sherif[1,2], Debbie C. Hocking[1], Susan Rees[3], Letizia M. Affaticati[1,2,4], Suresh Sundram[1,2,5*]*

Author Affiliations:

1. Department of Psychiatry, School of Clinical Sciences, Faculty of Medicine, Nursing and Health Sciences, Monash University, Clayton, Melbourne, Victoria, Australia.
2. Cabrini Outreach, Asylum Seeker and Refugee Health Hub, Melbourne, Victoria, Australia
3. Department of Psychiatry and Mental Health, School of Clinical Medicine, Faculty of Medicine and Health, University of New South Wales, Sydney, New South Wales, Australia
4. Department of Medicine and Surgery, University of Milan Bicocca, via Cadore 38, 20900 Monza, Italy
5. Monash Health, Monash Medical Centre, Clayton, Melbourne, Victoria, Australia

*Corresponding authors
Email: or Bafreen.sherif@monash.edu or suresh.sundram@monash.edu

Abstract

Background

Globally, the population of refugees and asylum seekers has reached unprecedented levels, prompting several host countries to implement immigration detention as a strategy to mitigate the influx of unauthorised arrivals. Previous research focusing on asylum seekers and refugees has predominantly utilised quantitative methods to assess the effects of immigration detention on mental and physical health outcomes. This study represents the first qualitative systematic review aimed at evaluating and synthesising qualitative research concerning the impact of immigration detention on the physical and mental well-being of adult and child asylum seekers and refugees (ASR) residing in middle- to high-income host countries.

Methods and findings

Between December 2021 and October 2024, ~~across~~ medical, allied health, and social sciences databases and grey literature (Embase, MEDLINE, PsychINFO, CINAHL Plus, Global Health, APA PsycARTICLES, Social Policy and Practice, Cochrane Library, Scopus, Open Grey)~~.~~ were systematically searched to identify original peer-reviewed articles describing qualitative findings relating to the experience of immigration detention among ASR in middle—to high-income countries. The included studies' references were also perused. The systematic review was registered with PROSPERO (CRD) 42022328867. Study selection, critical appraisal and data extraction were performed employing the Joanna Briggs Institute Critical Appraisal Checklist for Qualitative Research. Two reviewers identified relevant abstracts and articles. Synthesis of the included studies identified prominent themes relating to the effects of immigration detention on health outcomes in ASR adults and children. A total of 2,512 records were initially identified and abstracts reviewed, of which 564 full-text articles were assessed for eligibility. Twenty were included in the final review. The duration of detention ranged from 2 weeks to 38 months. The pooled studies included 374 adult asylum seekers and refugees and 139 children with a current or past history of immigration detention. A total of five major themes influencing the physical and mental health impacts of immigration detention emerged from the synthesis. Three were relevant to the detention period: i) Human rights violations in detention, ii) Systemic failures of healthcare provision and resultant ASR vulnerability and deprivation, and iii) Negative health impacts of immigration detention. Two themes were relevant to the post-detention period: iv) changes in self-construct, relationships and worldview, and v) enduring impact on mental and physical health.

Conclusions

This comprehensive review highlights the uniformly negative and persistent outcomes associated with immigration detention on the health of ASR adults and children. Findings may usefully inform future health and human rights considerations of migration policy.

Keywords: Refugees; asylum seekers; immigration detention; physical health; mental health; qualitative research

Author Summary

Why was this study done?

- Globally, the population of refugees and asylum seekers has attained unprecedented levels, with some host countries resorting to immigration detention as a strategy to curb an influx of unauthorised arrivals.
- This is the first systematic review investigating qualitative evidence related to the mental and physical health of asylum seekers and refugees who have experienced current or past immigration detention in middle- to high-income host countries.

What did the researchers do and find?

- We performed a comprehensive literature search examining qualitative evidence related to the mental and physical health of adult and child asylum seekers and refugees with a current or past experience of immigration detention in middle-to-high-income host countries.
- We conducted the systematic review with rigorous adherence to standardised methodology and comprehensively articulated it, enhancing its future replicability and reliability.
- Notwithstanding the diverse qualitative methodologies employed in the included studies, coherent and integrated findings were extracted, facilitating unique insights previously unexplored.

- This systematic review and thematic synthesis highlight the substantial long-term effects that immigration detention inflicts on the psychological well-being of both adult and child asylum seekers and refugees, both during their period of detention and aftermath. Additionally, it advocates for further research to bridge the current gap in evidence regarding the physical health of refugees and asylum seekers who have encountered immigration detention.

What do these findings mean?

- There is a pressing need for additional research focused on the development and evaluation of interventions designed to support asylum seekers and refugees in navigating the substantial changes in their lives following the experience of immigration detention, with particular attention to their mental and physical health.
- The findings derived from this synthesis hold significant potential for health professionals and policymakers involved in immigration detention, as they may aid in guiding the future formulation of treatment protocols specifically designed to address the needs of refugees and asylum seekers.
- The predominant body of research has focused on populations of asylum seekers and refugees (ASR) in the post-detention context, as opposed to during their current detention at the time of recruitment. This methodological approach has the potential to introduce recall bias in their narratives and the ensuing synthesis of data.

1. Background

Unprecedented flows of asylum seekers and refugees have resulted in many industrialised countries introducing punitive policies in an effort to curtail entry [1, 2]. One strategy is the utilisation of immigration detention to facilitate the expulsion of irregular migrants who enter in an unauthorised manner [3-5]. Once apprehended, immigration detainees find themselves subjected to conditions that may have a detrimental effect on their overall health and which, in extreme cases, may lead to mortality [6]. Detainees may be subjected to verbal and physical maltreatment, family separation, restraints, solitary confinement, inadequate nutrition, overcrowding, poor sanitation, and poor air and water quality, among other substandard living conditions [2, 7-11]. Given the variance in detention systems across countries and the differing terminology associated with detainees in various studies, this review adopted an approach encompassing materials related to individuals detained within an immigration context identified as refugees or asylum seekers. The terms "asylum seeker" and "refugee" are frequently utilised interchangeably and are perceived as synonymous. These concepts are interconnected, as they both "occupy a space" within international law, situated between the principles of state sovereignty and humanitarian protection. Within the framework of international law, a refugee is typically defined as an individual actively seeking or enjoying asylum [12]. However, in a more stringent legal context, the term may be restricted to individuals who meet the criteria established by the 1951 Convention Relating to the Status of Refugees or its 1967 Protocol (collectively known as the '1951 Refugee Convention') and who have been duly recognised as such. Consequently, the term "asylum seeker," along with the concept of asylum, is broader and encompasses all individuals pursuing asylum [13]. We

refer to this collective group as ASR to acknowledge the interchangeability and lack of consistent use of the terminology in academic discourse.

1.1 The influence of immigration detention on physical and mental health outcomes

i) Adults

ASR populations experience higher levels of post-traumatic stress disorder (PTSD), anxiety and major depressive disorder (MDD) compared with the general population [14-17]. The detention environment may further aggravate mental disorders, as indicated by elevated rates of both physical and psychological health conditions, self-harm, and suicide attempts among immigration detainees [18, 19]. This is particularly notable among individuals who have encountered pre- and peri-migration trauma and persecution [20-22].

The structural mechanisms of immigration detention, including inadequate medical care and substandard living conditions, may undermine the health of detainees and establish obstacles to obtaining necessary medical attention [3, 9, 18, 21, 23-27]. The detention environment has also been associated with physical health problems in detainees, such as gastrointestinal issues, respiratory infections and musculoskeletal complaints [28]. Access to sufficient healthcare and medical services within detention facilities may lead to untreated or poorly managed medical conditions, culminating in deteriorated physical health outcomes [28]. Furthermore, individuals may continue to experience adverse health consequences even after their release from detention [3, 29, 30]. Detainees frequently endure persistent symptoms of mental disorders such as MDD, PTSD, and anxiety, which may impede their successful reintegration into society [3, 29, 30]. Following release from detention, social and economic challenges can further erode overall health and well-being [31].

ii) Children

Similar to adults, immigration detention has been found to have an adverse impact on the physical and mental health of children, with a deterioration in mental health shown to be directly proportional to cumulative exposure to stress and trauma [32]. Immigration detention is recognised as a significant adverse childhood experience, contributing to or exacerbating negative health outcomes among children and adolescents who arrived as asylum seekers [33-39]. Furthermore, immigration detention acts as a post-migration stressor, markedly increasing the prevalence of Post-Traumatic Stress Disorder (PTSD) and Major Depressive Disorder (MDD) in children [32, 40, 41]. In addition to the detrimental physical and mental health consequences associated with detention, research has substantiated the occurrence of neurodevelopmental and educational challenges [42-46] alongside adverse ramifications on family dynamics [10].

1.2 Literature Gaps

The prominent systematic reviews within this domain have utilised quantitative data to examine variances in symptom endorsement patterns, specifically anxiety, depression, and post-traumatic stress disorder among detained and non-detained adult or child refugees [18, 19, 43, 47-49]. Additional systematic reviews have further incorporated aspects concerning physical health and social functioning among confined asylum seekers in detention [47, 50], alongside identifying distinct detainee characteristics correlated with an elevated risk within immigration detention [51]. These characteristics include the duration of detention, pre-existing trauma, such as torture and sexual violence [52], pre-existing mental and physical health issues, and the inadequate healthcare and mental health services available within the immigration detention system. The existing reviews of qualitative studies have assessed

related subjects, such as the healthcare needs of migrants while detained in European immigration detention environments [53] and a meta-ethnographic approach evaluating the effects of immigration detention on the health and well-being of refugees and asylum seekers [54].

To the best of the authors' knowledge, no systematic reviews have been conducted regarding qualitative studies on the physical and mental health impacts of immigration detention on asylum seekers and refugees (ASR). Thus far, global research pertaining to the physical and psychological health of ASR who have experienced immigration detention has predominantly employed cross-culturally validated quantitative assessment tools. Nevertheless, it is conceivable that certain attributes of the immigration detention experience, along with its corresponding health effects, may not be sufficiently captured within the current diagnostic criteria or measurement instruments. To investigate this potential gap, we undertook a systematic review of qualitative studies that examine the physical and mental health of ASR populations within the framework of their immigration detention experience.

1.3 Aim and significance of this review

This systematic review was conducted to explore the qualitative literature describing the impact of immigration detention on the physical and mental health of adult and child ASR in middle- to high-income host countries. This is the first qualitative systematic review to assess physical and mental health associations with current/ prior detention in both adult and child populations.

2. Methods

2.1 Protocol and eligibility criteria

Systematic searches for peer-reviewed qualitative literature were conducted utilising the Joanna Briggs Institute (JBI) Checklist for Qualitative Research [55]. Studies were included if they met recognised qualitative reporting standards (Standards for Reporting Qualitative Research, SRQR) [56]. The review was carried out by four researchers (BS, DH, LMA and SS). The protocol for this systematic review was registered with the International Prospective Register of Systematic Reviews (PROSPERO), registration number CRD42022328867.

2.2 Inclusion and exclusion criteria

Qualitative or mixed-methods studies were included if they i) sampled adults or children detained for immigration purposes, either whilst detained or post-release from immigration detention, and ii) described physical and mental health outcomes as a consequence of immigration detention.

Studies were excluded on the following grounds: i) did not report specifically on the health impact of immigration detention; ii) detention exclusively had a criminal justice purpose; and iii) detention did not deprive the freedom of movement (e.g., migrant reception centres).

Qualitative or mixed-methods studies, ~~including~~ ASR, and observations of key informants, professionals or stakeholders working with ASR were included. No restrictions were placed on age, sex, detainee country of origin, or method of describing physical and psychological

health outcomes. No restrictions were placed on the publication date or language of publication.

2.3 Information sources and search strategy

Relevant studies were identified through electronic searches of Embase (1980 to 2024 week 43), OVID MEDLINE (1946 to 2024 week 43), APA PsychINFO (1806 to 2024 week 43), CINAHL Plus (1937 to 2024 week 43), OVID platform (Global Health (1910 to 2024 week 43), APA PsycARTICLES (1967 to 2024 week 43), Social policy and practice (from 1981 to 2024 week 43), Cochrane Library (from 1993 to 2024 week 43), Scopus, Open Grey and advanced Google search of .org websites until ten consecutive pages did not include relevant pages (searched 2024 week 29). A comprehensive search strategy was developed for Ovid Medline and then modified for other databases, utilising an earlier review by von Werthern et al. (2018) [57] as a template (see supplementary material 1). Electronic searches were supplemented with the screening of reference lists of included primary studies. All study titles and abstracts were imported into an Excel spread and stored by the primary author.

2.4 Study selection and data collection

Study title and abstract screening and full-text screening were done by the primary author against predetermined criteria, with three additional reviewers (DCH, LMA, SS) providing input where any disagreements arose. Relevant data were extracted and entered into an Excel sheet by the primary author, accuracy-checked and consolidated independently by three reviewers (DCH, LMA, SS), with a fourth reviewer (SR) providing overarching support and consolidation. We extracted data pertaining to general study characteristics and

methodology, participant demographics and qualitative content, and detention duration where stipulated, which is described in detail in Tables 1 and 2.

2.5 Quality Assessment

Quality was assessed using the Joanna Briggs Institute (JBI) critical appraisal tool [55]. Studies were included without regard to their methodological quality, as the insights gathered from study participants were deemed valuable for our synthesis [58]. This consideration reflects the innovative character of this qualitative systematic review, which aims to encompass a comprehensive array of literature pertinent to the research question. Among our findings, we address the limitations observed in the included studies.

2.6 Summary measures and synthesis of results

Results were analysed using a textual narrative synthesis approach, as described by Lucas et al., [59] in which individual study characteristics and findings are reported according to a standard format, and structured summaries are developed to elaborate on and contextualise the data extracted across studies [59, 60]. The qualitative context extracted included child or adult ASR participants or observations of key informants, professionals or stakeholders working with ASR either during or in the aftermath of the immigration detention experience and described the physical and/or mental health outcomes as a consequence of immigration detention. Qualitative data extraction was performed by the primary author (BS) and entered into an Excel spreadsheet. Following initial data extraction, synthesis of findings from individual studies was done by (BS) with input from study authors (DCH, LMA, SS, SR) focusing on the scope, differences and similarities among studies to make broader inferences about the impact of immigration detention on the physical and mental health outcomes of adult or

child ASR [59]. Data domains were grouped in relevance across respective themes, which were agreed upon by the study researchers.

3. Results

3.1 Search results

Out of 2512 individual records identified, 20 studies were eligible for inclusion – Figure 1. Of the 20 studies included, seven were conducted in Australia [61-67], four in the United Kingdom [68-71], three in Canada [72-74], three in the USA [2, 75, 76], one in Serbia [77], one in Sweden [78] and one in the Netherlands [79]. Of the studies, six were published in the last five years. Table 1 presents the characteristics of the included studies. Studies were conducted in high-income countries, with only 1 study reported in a middle-income country.

3.2 Study characteristics

Study characteristics are summarised in Table 1. A total of 513 participants (374 adults and 139 children) were included across the studies, comprising refugees (N=222), asylum seekers (N=153), and combined ASR (N=138). Participants originated from North Africa, Middle Africa, East Africa, West Africa, East Asia, Central Asia, South Asia, West Asia, Europe, North America, Central America, South America and the Caribbean. Fifteen studies focused exclusively on adult ASR [2, 3] [61, 70, 72, 80] [65-69, 71, 75, 78, 79], three on children and families [73, 74, 81] and one on adolescents [76]. One study focused on adult male undocumented migrants [77].

Three studies included observations of the health of ASR by health professionals [70], health and social service workers [61] and detention centre staff (mental health, welfare or religious

services) (n=11) [71]; two studies related to detainee self-harm and suicide in detention [71, 75]; one study included four migrant women who had been held in detention while pregnant [70]; and one study included 9 ASR patients who had been treated for PTSD at a specialist mental health service and had previously been in immigration detention [79].

3.3 Quality Assessment

The JBI was used to characterise the strengths, limitations, and impact of pertinent data in addressing the research objectives and the quality of evidence across the included studies [55]. Supplementary File 2 provides an overview of each study.

Among the 20 studies included in this review, nine met the criterion for congruence between their philosophical perspective and research method (criterion 1). In comparison, the remaining eleven studies did not state their philosophical or theoretical premises. Eighteen studies demonstrated congruity between their research methodology and the research question or objectives (criterion 2). The same 18 studies also demonstrated congruity between their methodology and the methods used to collect data (criterion 3). The latter two criteria were unclear in two papers due to insufficient information about the methodology used. Seventeen studies showed congruity between methodology and data representation and analysis (criterion 4); this criterion was unclear for one study due to inadequate description of data analysis and for two studies due to insufficient detail in methodology. For all 20 studies, there was congruence between methods and interpretation of results (criterion 5). Only four studies reported a statement locating the researcher culturally or theoretically (criterion 6); the influence of the researcher on the research and vice-versa (criterion 7) were addressed in two of the included articles. In all studies, we determined that participants and

their voices were adequately represented (criterion 8). This last criterion did not apply to the remaining study due to the research method, which involved the analysis of secondary sources. Ethical approval by an appropriate body was reported in 15 studies (criterion 9). Three studies did not provide such statements, and the criterion was not applicable to two studies due to the type of data sources utilised. Conclusions were appropriate for 19 papers; for one study, conclusions were deemed too partial (criterion 10).

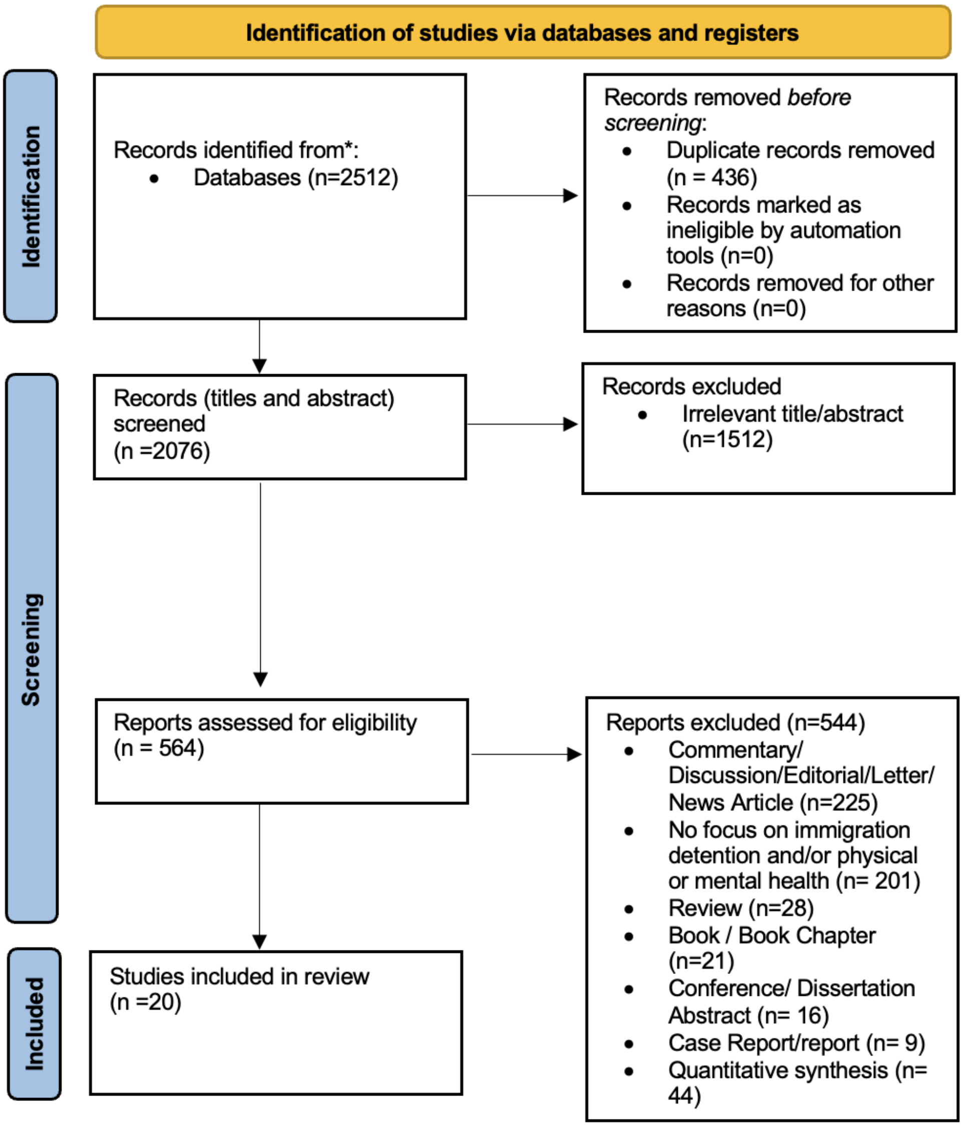

Fig.1 PRISMA Flow diagram for the systematic review of qualitative studies

Table 1 Characteristics and key findings of the impacts of immigration detention on asylum seekers/ refugees (n=20)

Author and year	Phenomena of interest	Sample and participant characteristics	Country of origin	Country of study	Method for data collection and analysis	Description of main results
**Arsenijevic et al, (2018) [77]**	Self-perceived vulnerabilities of male migrants travelling alone along the Balkan route to Europe.<br><br>Detention duration not specified	30 adult male migrants travelling along the Balkan route	Afghanistan, Pakistan, Algeria, Iraq, Syria, Morocco	Serbia	Individual interviews, group interviews and participant observation.<br>Data was analysed using thematic content analysis.	Participants describe the precarious living conditions in most of the detention camps, including the overall lack of food, extremely poor sanitary conditions, overcrowding and high incidence of diseases, and limited or no access to medical care. There was an instance of a detention camp where participants were reportedly kept in cages, in inhumane and undignified living conditions. Dire mental health conditions of co-detainees were also described.
**Arshad et al (2018) [70]**	Pregnant migrant women's experiences of living in detention.<br><br>Detention duration: 53- 212 days	4 migrant women who had been experienced some/all of their pregnancy while in detention; two volunteer health professionals	Central Europe, Sub–Saharan Africa and India	UK	Semi-structured interviews. Data was thematically analysed using Braun and Clarke's six-stage approach.	Pregnant migrant women in detention are noted to be a vulnerable group with poor underlying mental and physical health that is exacerbated by the detention process. Four key themes emerged: 1) challenges in accessing maternity care, 2) exacerbation of mental health conditions, 3) feeling hungry, and 4) lack of privacy. Results suggest that women face significant challenges in the access of timely and appropriate maternity care, their health concerns are disregarded and they experience a lack of continuity of care.
**Boerma et al., (2022) [79]**	Experiences of adult refugees who had been held in immigration detention in the Netherlands and the impact of this on their mental health.<br><br>Detention duration: Range 19 – 365 days.	9 adult ASR (7 M, 2 F); age range 19-44 years) who were treated for post-traumatic stress disorder at a specialist mental health facility and who had previously been in immigration detention	Sub-Saharan Africa, Central Asia	Netherlands	Semi-structured individual interviews guided by a topic guide.<br><br>Data was analysed using grounded theory with codes being grouped and merged, later utilising axial coding.	All participants reported that their experiences in immigration detention were significantly impactful and detrimental to their mental health. The interviews revealed the following primary themes: uncertainty regarding expectations, the perception of being treated as criminals, anticipation of protection upon arrival in the Netherlands, and the experience of re-traumatization. These perceptions engendered feelings of anxiety, hopelessness, shame, anger, and suicidal tendencies. A significant number of participants were subjected to isolation, which exacerbated their mental health conditions.
**Campbell and Steel (2014) [61]**	Experiences of asylum seekers detained in Australia to explore the relationship between mental wellbeing, living conditions, and Australia's detention policies.<br><br>Detention duration not specified	7 adult asylum seekers (6 M, 1 F) who had previously lived in a range of Australian detention facilities;3 health and social-work professionals	Sri Lanka, Iran, Iraq, Afghanistan, Pakistan	Australia	Ethnographic observations, semi- structured interviews, key-informant interviews, and document analysis of international human rights protocols.<br>Data was analysed using Charmaz's methods for constructivist grounded theory.	Results highlight the mental distress suffered by asylum seekers in detention, the environments of constraint in which they live, and aspects of detention centre policy that contribute to these environments. A discrepancy between asylum seekers' experiences under immigration detention policy and Australia's human rights obligations is also reported.
**Cleveland et al, (2018) [72]**	Adult asylum seekers' experience of detention in	81 adult asylum seekers held in two Canadian immigration detention	Sub-Saharan Africa, South Asia, Middle East and North	Canada	Semi-structured interviews. Data was analysed using thematic content analysis.	Detention, even for brief periods in relatively adequate conditions, was found to be detrimental to asylum seekers' mental health. This adverse impact appears to be largely attributable to the combined

	Canadian immigration detention centers<br><br>Mean detention: 31 days	centers at the time of the study.	Africa, Latin America, Caribbean, Europe.			effect of two factors: symbolic violence and disempowerment. Asylum seekers express shock and humiliation at being ‘‘treated like criminals.’’
**Coffey, (2010) [3]**	Experience of extended periods of immigration detention from the perspective of previously detained asylum seekers and consequences of these experiences for life after release<br><br>Mean detention: 3.17 years	17 adult refugees (16 M, 1 F) who had been held in immigration detention funded by the Australian government. Participants were living in the community and had been granted permanent visa status or such status was imminent at the time of the study.	Afghanistan, Iran, Iraq, Other Middle-Eastern	Australia	Semi-structured interviews. (on average three years and eight months following participant's release from detention). Data was analysed using thematic coding.	Participants struggle in rebuilding their lives in the years following release from immigration detention, with the majority experiencing pervasive difficulties. Participants endure an ongoing sense of insecurity and injustice, struggles with relationships, profound changes to view of self and poor mental health. Depression, demoralisation, concentration and memory disturbances, and persistent anxiety are commonly reported.
**Diaz et al, (2022) [2]**	Impact of the US immigration detention system on the health of people recently released by ICE.<br><br>Median detention: 11 months	16 adult migrants (13M, 2 F, 1 non-binary) released from US immigration detention within two years of study enrolment	Dominican Republic, Jamaica, Trinidad and Tobago, Honduras, Mexico, Guatemala, Pakistan, Russia, Kazakhstan	USA	Semi-structured anonymous phone interviews. Data was processed using reflective thematic analysis with an inductive approach.	Four themes emerge from analysis: 1) poor conditions and inhumane treatment, 2) a pervasive sense of injustice, 3) structural barriers limiting access to care, and 4) negative health impacts of immigration detention.
**Hollis, (2018) [68]**	Explore the psychosocial stressors experienced by people in detention, the psychological impacts of being detained and the ways in which people express resilience and cope in detention.<br><br>Mean duration: 3 months (Range 24 hours- 7 months)	9 adult asylum seekers (7 M, 2 F; mean age 31) who had previously been held in UK IRCs.	Iran, Iraqi-Kurdistan, Pakistan, Yemen, Zimbabwe	UK	Semi-structured interviews conducted guided by a topic guide.<br><br>Data was analysed using interpretive phenomenological analysis.	Participants exhibited incredulity and cognitive dissonance regarding their detention, resulting in their deprivation of communication and healthcare. These stressors culminated in feelings of powerlessness, self-doubt, and contemplation of their uncertain futures. Nevertheless, participants demonstrated resilience by adopting proactive behaviours, engaging in spiritual practices, and relying on personal relationships to cope during their time in detention.
**Johnston, et al (2009)[80]**	Association of Australian asylum policies and practices with adverse health outcomes<br><br>Mean detention duration <12 months	60 adult refugees with Temporary Protection Visas (TPV) living in the community, with a previous experience of immigration detention	Iraq	Australia	Semi-structured interviews.<br><br>Data was analysed using thematic coding.	Participants generally feel socially isolated and lacking in control over their life circumstances, because of their experiences in detention and on a temporary visa. Perception of injustice in immigration detention is a significant cause of anger, with many feeling that that detention policies violate their human rights. The disrespectful treatment of women, lack of access to female doctors for Muslim women, and the overall loss of freedom are highlighted as central concerns in immigration detention.

**Kellezi & Bosworth, (2016)[71]**	Mental health, self-harm and suicide in detention (UK immigration removal centers, IRC).<br><br>Detention duration not specified	19 adult migrant women detained in an IRC at the time of the study; 6 members of staff	Not reported	UK	Semi-structured interviews.<br><br>Details of data analysis not reported.	Most detainees report very high levels of anxiety, distress and depression. Nights, early days in detention, negative immigration outcomes, concerns about medical and physical health, witnessing other detainees in distress or being removed, and separation from family (especially children) are described as particularly difficult.
**Kronick et al, (2015) [82]**	Experiences of detained children and families who have sought asylum in Canada.<br><br>Mean detention duration 56.4 days	20 families (65% asylum seekers, 35% failed refugee claimants) comprising of 35 children in total. 12 families were interviewed during detention and 8 families post release from detention.	Europe, Southwest Asia, West Africa, East Africa, North America, Middle east, Central Africa, South Asia, Latin America	Canada	Semi-structured interviews and ethnographic participant observation.<br><br>Data was analyzed through inductive coding guided by thematic analysis principles.	Detention is described as a frightening experience of deprivation leaving children feeling criminalized and helpless. Family separation is noted to further shatter children's sense of well-being. Children's emotional and behavioural responses to separation and to detention indicates experiences of acute stress and, in some cases, trauma. Even when detention is brief, distress and impairment often persisted months after release.
**Kronick et al, (2018) [74]**	Lived experiences of children held in Canadian immigration detention.<br><br>Mean detention duration: 90.4 days	10 children from 8 different families, either living in detention or with an experience of immigration detention at the time of the study. (Mean age: 8.5 years; range 3-13)	Not reported	Canada	Sand-play and narrative inquiry – data included photos and drawings of sand-trays, interviews with parents and key informants, transcriptions of sand-play stories, field notes. Data was analysed using coding themes for sand-trays and sand-play stories, and re-contextualized in data gathered from field notes and parents' interviews.	The interpretation of children's oral narratives and symbolic worlds generates three broad themes: 1) confinement and surveillance; 2) loss of protection; and 3) human violence.
**Mares & Jureidini, (2004) [81]**	Mental health (of children and families living in immigration detention<br><br>Mean detention duration: 1.25 years (range 12-18 mo)	16 adults (7M, 9 F) and 20 children from 10 families who, at the time of the study, were being detained in a remote Immigration and Reprocessing Centre (IRPC) and had been referred to a specialist mental health service.	Iran, Iraq, Afghanistan, Palestine	Australia	Multiple, detailed clinical interviews, undertaken by a range of experienced mental health clinicians at a specialist mental health service,<br><br>Details of data analysis not reported.	There are high levels of psychiatric distress among both adults and children. All children had at least one parent with psychiatric illness, with the majority of adults meeting criteria for MDD and PTSD. Children under five showed developmental delays and attachment disturbances; all had been exposed to violence and chronic parental mental illness. Older children exhibit PTSD symptoms related to traumatic experiences in detention, including witnessing severe self-harm and suicidal behaviour. They struggled with sleep, concentration, and feelings of hopelessness, and reported anxiety about parental well-being. Parental incapacity due to mental illness often left children assuming caregiving roles, exacerbating their emotional burden.

**Marquez et al (2021)[75]**	Policies and prevention measures for detainee suicides within Immigration Customs Enforcement (ICE) detention facilities<br><br>Detention duration not specified	N/A	Latina American, other (not reported)	USA	Inspection reports detainee death reports , and intergovernmental contracts from 116 large ICE detention facilities<br><br>Data was analysed using thematic coding with an inductive approach.	Detention facilities were noted to be inadequately staffed and resourced, resulting in the failure to implement federally mandated protocols regarding detainees' well-being competently and promptly.
**Passardi et al, (2022)[65]**	Moral injury appraisals and associated mental health outcomes related to immigration detention on Nauru.<br><br>Detention duration: 14.2 months (SD = 11.1)	13 adults who had sought (9F, 4M) refuge in Australia and, due to arriving by boat, had been transferred to immigration detention on Nauru. Participants were living in Australia for 28 to 64 months following their medical evacuation (M= 55.3, SD= 12.3) and had spent between 1 and 36 months in Nauru (M= 14.2, SD= 11.1).	Iran, Somalia, Nepal, Sri Lanka, others	Australia	Semi-structured in-depth interviews.<br>Data was analysed using reflexive thematic analysis.	Participants met the criteria for PTSD (n=10), Major depressive disorder (n=9), panic disorder (n=3), and obsessive-compulsive disorder (n=3); only two had no current disorder(s). Four participants expressed suicidal ideation, three had past suicide attempts more than two years prior, and one participant had made a suicide attempt less than two years prior. Major themes included participant experience of deprivation, lack of agency, violence, and dehumanisation after arrival, with the Australian government seen as the driving force behind these experiences. These experiences culminate in feeling irreparably damaged for ASR with an experience of detention.
**Puthoopparambil et al, (2015) [78]**	Perceptions and experiences of immigrant detainees in Swedish immigration detention centres.<br><br>Detention duration: 2 weeks – 9 months	21 adult immigrants (19 M; 3 F) detained in three Swedish immigration detention centres at the time of the study.	Belarus, Chile, Guinea, Iraq, Morocco, Nicaragua, Palestine, Senegal, Serbia, Sierra Leone, Uganda and the USA	Sweden	Semi-structured interviews<br><br>Interview transcripts were analysed using thematic analysis (Braun and Clarke, 2006)	The detainees liken immigration detention to imprisonment. There is a lack of control over their life mainly due to arbitrary restrictions and inadequate response from authorities, which make seeking help appear futile. This perceived lack of control forced them into passivity. Differences in amenities provided in different centres were observed and some of these were reported to help in making detention more bearable.
**Rothe, (2003) [76]**	Psychopathology of refugee adolescents confined to detention in Guantanamo Bay.<br><br>Detention duration: 6-8 months	74 adolescents (47 M, 27 Female; mean age 15.5 years) who had left Cuba by sea en route to the USA but who were intercepted and confined to detention in Guantanamo Bay, where they were held at the time of the study and referred to a psychiatric clinic for evaluation. 66% of the boys and 70% of the girls were	Cuba – ethnicity not specified	USA (Guantanamo Bay)	Clinical interviews, including projective drawings<br><br>Details on data analysis not reported.	Notable themes developed included: evidence for pre-existing vulnerability to trauma; presence of post-traumatic and regressive symptoms and behavioural problems; interaction of these symptoms with lives in the camp; experiences of dehumanization inside the camps.

		accompanied by both parents.				
**Shishehgar et al, (2021) [66]**	Experiences of asylum seeker women living in Australian immigration detention and impact on mental health.<br><br>Detention duration: 1-4 months (range)	17 adult Iranian female asylum seekers, previously detained and living in the Australian community for 2-3 years on bridging visas at the time of the study.	Iran	Australia	Semi-structured interviews<br><br>Data was analysed using thematic and inductive analysis.	Living in a prison-like environment was described as a punishment for their boat arrival, violating their privacy and dignity. In contrast, a sense of security, free access to healthcare services, and the possibility to build social networks were positive aspects of short-term detention.
**Witney & Bates, (2016) [67]**	Experiences of immigrants previously held in Australian immigration detention and the incorporation of their traumatic experiences within the self-construct<br><br>Detention duration >2 years	10 adult male former asylum seekers living in the community on TPV and permanent protection visas (PPV) after being held in mandatory detention ; 10 males age 19-51.	Not reported	Australia	Semi-structured interviews. Data was analysed using thematic analysis.	Before receiving their visas, all participants had been held in detention centres onshore on the Australian mainland (n=7) or offshore in neighbouring Pacific islands (n=3) for periods ranging from 3 to 7 years. Interviews occurred around six months post-release from detention into the community. Thematic analysis revealed that participants experienced similar levels of narrative disturbances as a result of detention, which affected their sense of past, present, and future. However, their subsequent narrative integration varied. Three groups representing different levels of narrative integration were identified: robust integration, limited integration, and fragmentation. Higher levels of integration were linked to a greater sense of unity and purpose in their stories.
**Zimmerman et al., (2012) [69]**	Exploratory study seeking to test the usefulness of a questionnaire among former immigration detainees examining perceptions of their health needs and access to care within immigration detention in the UK.<br><br>Detention duration: median 185 (IQR: 10-990) day	30 adult participants (26 M, 4 F; mean age 33) of whom 27 were asylum seekers and 3 were detained on judicial grounds (without seeking asylum thus excluded). All had been released from detention within the past 12 months.	Afghanistan, Iran, Nigeria, Somalia, Morocco, Zimbabwe, Palestine, Benin, Ghana	UK	Semi-structured interviews guided by a interview topic guide.<br><br>Data was analysed using thematic analysis.	The findings indicate that 66 percent of participants entered detention with pre-existing mental or physical health conditions necessitating ongoing or new treatments. Furthermore, the majority of participants experienced the emergence of new mental/emotional (93 percent) or physical (53 percent) health issues during their detention. Access to healthcare within the detention environment was frequently hampered by various formal and informal challenges, affecting both its effectiveness and availability. The authors conclude that this exploratory study raises significant concerns regarding the adequacy of mental and physical health care provisions for detainees within the current UK detention system.

Note. ICE = Immigration Customs Enforcement; IRC = Immigration Removal Centres; PPV = Permanent Protection Visa; TPV = Temporary Protection Visa

3.4 Synthesis findings

Five major themes (Figure 2) were identified through the qualitative synthesis: three themes were relevant to the detention period (Themes 1-3), and two concerned the post-detention period (Themes 4-5). Table 2 references supporting quotes from the included studies within their respective themes.

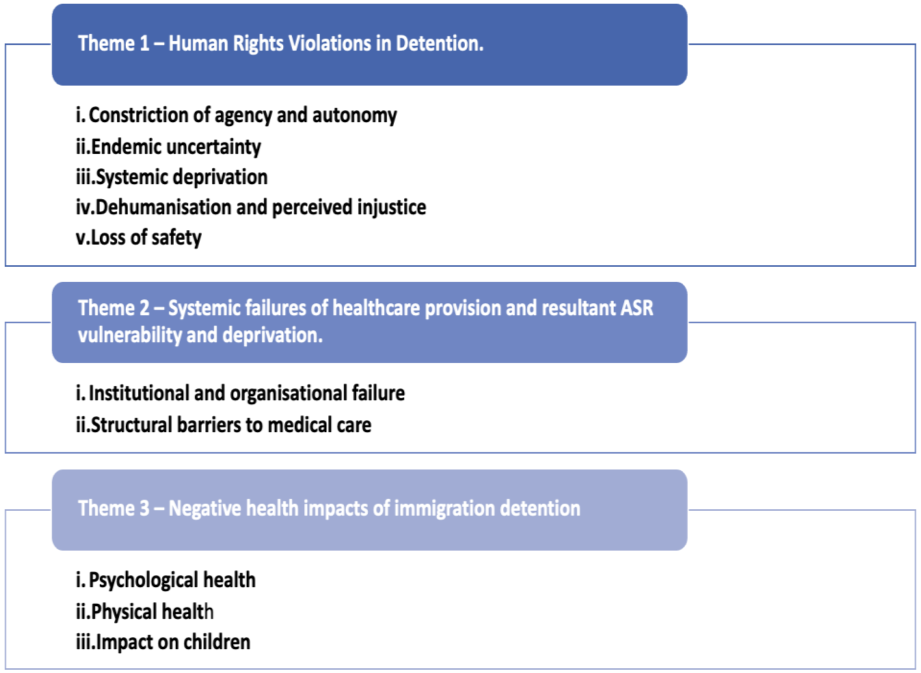

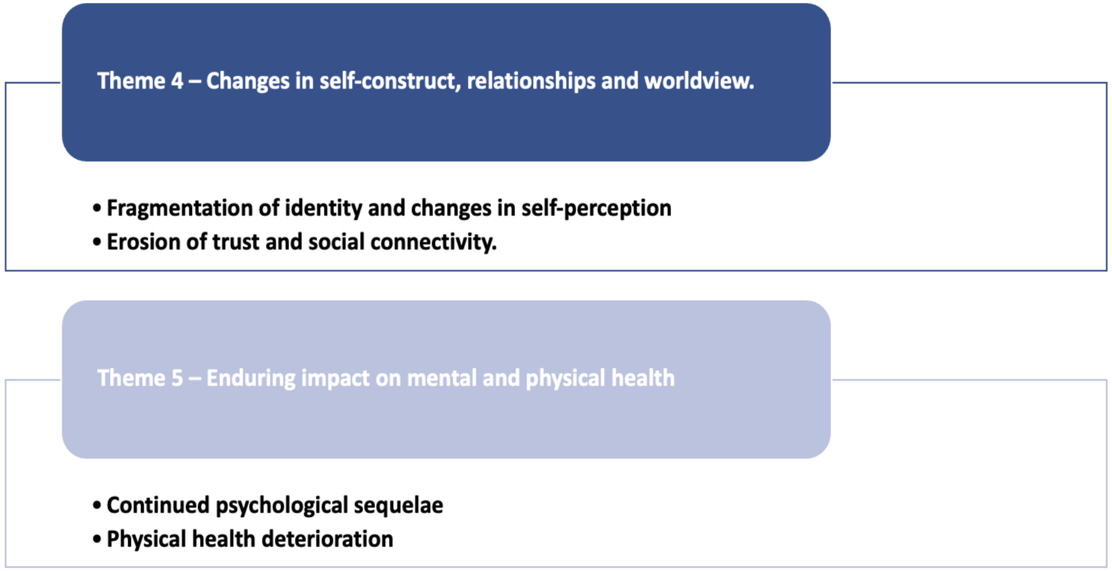

Figure 2 –Thematic Qualitative Synthesis

Table 2 Selected Supporting Quotes from Studies

Themes and sub-themes	Supporting quotes	Contributing studies
**Detention Period**		
**Human Rights Violations in Detention**<br>• Constriction of agency and autonomy<br>• Endemic uncertainty<br>• Systemic Deprivation<br>• Dehumanisation and perceived injustice<br>• Loss of safety	• “.. just being isolated, no privacy, men walking in ... feeling powerless….I couldn’t stop crying. I didn’t expect this, after what I had just been through in my country.’’<br>• “It is like you are a big criminal, you are there even though you never did any crime, or you never did anything wrong…but they are watching you.,,every step you take from outside your room…. If they make all the walls or fence with gold, there is nothing different, there is nothing changed, prison is prison. Still this system keeps me in detention for no reason.”<br>• “There is no justice in detention in the eyes of the detainee; some people are released very quickly, some may stay there like I did for more than two years, and some may be there for five years.”<br>• "I didn’t even know [where I was]. Because they didn’t even tell me. They just brought a letter for me. And it was in Dutch also. I have to sign this letter. Okay."<br>• “you’re locked up in a box... you don’t even get sunlight. You’re begging for sunlight, imagine that.”<br>• "I didn’t do nothing and they handcuffed me and [...] when I reached there, I want to ask like: 'I want to pee first' and there it was a police eh..., with me in the toilet. I don’t know the reason why."<br>• “The first night in detention I can never forget. When I was put in a single cell with no food. And I was hungry and lonely. Because this happened in a country where there should be human rights and respect for human rights. I can never forget that.”<br>• “This is such an unfair policy, especially for children. They come here looking for a safe place; they are not criminals. We are just normal people looking for a safe place...There are no human rights coming to us here...We are all people looking just for freedom but when they put us in this jail, they take the most important thing for us; our freedom.”<br>• “It’s like a prison... My dad was living in another building. He was separated from us.... Here, this guy was watching us so we could not go outside... These guys were watching us not to flee”<br>• “‘Life is like ... we are like garbage. A dog is better than a man… they put 30 of you in a room like cows, so how can you sleep?”<br>• “We were kept in a prison, given a room in the camp. It looked like a cage ... Officers entered occasionally into my room without knocking the door ... I wanted to go back to Iran from the Christmas Island detention.”<br>• “They took us to another camp, made of fences, like cages. They put 40–50 men in closed fenced cages. It was like a really big hall, closed, and inside that, there were fences. They gave us some food in the morning and evening, a really small piece, just so that you can stay alive with it. Just once in 24 hours you were allowed to go to the toilet and there were no facilities to take a shower... The fences are locked. You are not able to go out. The living conditions were really bad, really terrible..”<br>• “The red light in the room at night, its . . . to see if all detainees are in the room . . . I mean, they follow you like . . . like criminals. So, they do not speak to you, they watch you.”	Arsenijevic, 2018<br>Arshad, 2018<br>Boerma, 2022<br>Cleveland, 2018<br>Coffey, 2010<br>Diaz, 2022<br>Hollis, 2018<br>Johnston, 2009<br>Kronick, 2015<br>Kronick, 2018<br>Passardi, 2022<br>Puthoopparambil, 2015<br>Shishehgar, 2021

	• “We feel as an animal. It’s not what they do to humans. That time, I felt they were looking at me as an animal. But when they transferred me to Australia, I thought, no, I thought wrong because the people have a very nice behaviour with pets. When I went to the shop for the first time, I saw lots of food for dogs or cats. They have got toilets, they have got doctors. They have everything they need. So, we are smaller than that for the people who work in the detention camp or government.”<br>• “One behaviour from the officers that we didn’t really like and we actually wrote complaints about that. We found it very assaulting and insulting when they used to stand behind the fences and throw bags of clothing to us without even coming over as if we’re ill or sick people or have got something contagious . . . You know, all of us, my friends, my family and I, were so upset about that. We were saying “Do we have a skin condition that you just don’t want to be in contact?”’	
**Systemic Failures of Healthcare Provision and resultant ASR vulnerability and deprivation**<br>• Institutional and organisational failure<br>• Structural barriers to medical care	• “I would like to see the midwife because I would like to know what’s happening … I just want to hear my baby heart beat ... so I would wait all day but the midwife never come back to see me.”<br>• “Before I saw the doctor – he did not help me for the pain of my tooth. And the security guard helped me and I took one of my teeth out myself.”<br>• “I’d write on the screen, I need to talk to the psychologist, please, it’s urgent, I feel depressed, please help me... when you write sometime like that it’s because you really have a serious problem... 2-3 weeks later they’d say, okay, come over here.”<br>• “In fact, the doctor told me pretty much, “’you’re not dead yet, so there’s no reason for us to help you yet.”<br>• “We went to the labour ward ... the midwife ask the guards to leave because she need to check me ... the guard ask her to “leave the door open because we need to know what’s happening.”<br>• “Because I’m not criminal. (...) I was shamed to go to hospital because people are thinking ‘Who is he, has he killed somebody here? (...) You know, when I come to this country, I come for the freedom. I was shamed to go to hospital with [shackles], I refuse them.”<br>• “They disinfected everything, they wore gloves. Instead of handing me my food tray, they threw it on the table, as if I was an animal. (...) They didn’t respect me. I asked for help and that’s how they treated me.”<br>• When I saw myself in the mirror, it gave me a very bad feeling about myself – about my health, my back. And no one helped. I was so sad because of this.<br>• “I asked the nurses – I said I want to see the GP several times and they kept saying ‘‘we’ll call you’’. For almost 2 and a half months. There was no way. Just paracetamol is the only painkiller. You had to realize it was enough if you couldn’t get anything else.”<br>• After release from detention doctors outside ‘‘started listening. The ones there weren’t listening. Now they ask about your previous life and what makes you upset – they treat you like a person. In detention they just give tablets’’	Arsenijevic, 2018<br>Arshad, 2018<br>Campbell, 2014<br>Diaz, 2022<br>Hollis, 2018<br>Zimmerman, 2012
**Negative health impacts of immigration detention**<br>• Psychological health<br>• Physical health<br>• Impact on children	• “As you can see, I have very high blood pressure. Everything is affecting me badly. Here we are stuck between life and death. Our life is ahead of us. Future, to go out ... to go home, go back and die. ... I am full of regret. Full of regret for my whole life. I wish I had never left. I came here and there’s nothing for us, there is no one to help us.”<br>• "It was like, you know, sometimes if you have a wound. And someone is still peeling it again. [...] I have a wound. And they’re still adding more pain on me, again."<br>• "Because in my mind, I was asking myself: what, what’s going to happen to me? [...] You know, sometimes in Africa, they kill people. Like how they kill them, is like cutting the part. They cut your hand. They cut your arm. Cut your finger. They cut you. They kill you in pain before you die."	Boerma, 2022<br>Campbell, 2014<br>Coffey, 2010<br>Hollis, 2018<br>Mares, 2004<br>Passardi, 2022<br>Shishehgar, 2021

	• "Most of the time, I just took the tablets, and I was unconscious on my bed, sleeping. I rarely ate lunch. [...] When I saw myself in the mirror, it gave me a very bad feeling about myself – about my health, my back. And no one helped. I was so sad because of this."<br>• "I had very severe morning sickness. Very severe, you cant imagine. I couldn't go for six months in dining room. I never eat food, for six months of pregnancy. It was, just, orange in a whole day, one orange. Sometimes nothing. I cried for plain rice. Can I eat? But, I couldn't, I couldn't. They never allow the food in the room. [...] They just say no, it's rule. They couldn't give you food in the room."<br>• "It's the surroundings. It's what you're in. You are in a place with 250 other people – and of the 250, there's 10 happy ones. And the rest are just really at various stages. From really desperate, to suicidal, to depressed. It's not a place to be for any length of time."<br>• "Because the situation was so, so hard, that the Iranian boy – he hanged himself by rope. To kill himself. But he wasn't successful. The situation then was so, so horrible.<br>• "If I think of the places in detention centres where I was, I feel very upset and scared because I was not happy at all in there. [...] if I think of it I have the feel of shivering. And also, when I was in the detention I thought will I do something to harm myself, that how much I was upset and stressed out"<br>• "If you were very strong, a strong, strong human, they put you in isolation.one day, two days, one month, two months. This makes you damaged. This makes you crazy. You just go into the blanket, and lower, and lower. And you are thinking under the blanket."<br>• "The worst thing was that day by day there was no answer; then there was no hope… For each rejection, I used to [go] into my room for one week. [I] couldn't eat and [I had] a really strong sense of hopelessness. I was so tired, so scattered."<br>• "In my country they torture your body but in Australia they kill your mind"<br>• "Living in detention, it's like looking at oneself in a mirror. When I saw someone try to commit suicide; it's exactly like I'm doing that to myself."<br>• "..for 4 days I couldn't eat. I cried every day from morning to night. Yeah, I think he [my infant] became sick because he was away from his mother. He cried every day [too]."<br>• "'N', 'aged 12, drank coffee in an attempt to remain awake all night for fear that his depressed mother or psychotic father (whom he had witnessed dancing naked in the camp) might come to grief without his vigilance. He had been victimised by other detainees and guards because of his father's bizarre and provocative behaviour."<br>• "We [siblings] said we are together, but they [detention officers] told us 'here is Australia, sisters and brothers are not part of family. You and your husband and your child are a family' ... My sister was detained in the single detention ... I am very dependent on my sister. It was important for me to be with my sister."<br>• "You know you can't throw [away] your own background...the greatest torture of all is not being able to contact your family members and not being able to tell them what is happening to you."	Witney, 2016
**Post-detention period**		
**Changes in Self-construct, relationships and worldview**<br>• Fragmentation of identity and changes in self-perception	• "Detention has the effect that it makes you a person who becomes passive, lazy . [It] pacifies a person, their personality, and stops people from actively pursuing their goals."<br>• "As a normal human being I don't have the courage to talk to people, normally. After four, five minutes my entire body will be shaking or shivery."<br>• "At the moment my relationship with [my family] is fine, but they don't know what's in me. I won't tell them because they have suffered enough so there is no point to let them share my pain. Secondly, I want them to go on with their future, and I want them to be far from these issues."	Coffey, 2010<br>Kronick, 2015<br>Witney, 2916

• Erosion of trust and social connectivity.	• "I did not attend [English classes] because I believed that we will not be released from the detention centre and I thought 'What for?'"<br>• "I'm constantly aware of my actions because I'm afraid that if I do any slight thing wrong they might tell me that "you need to go back to detention".'	
**Enduring impact on mental and physical health**<br>• Continued psychological sequalae<br>• Physical health deterioration.	• "But how is it possible one person should be in detention [for] five years? I missed my life. My children didn't have a father, and my wife didn't have a husband. It's just not right."<br>• "I worry about my future. I am now 39 years old. The train I might have caught has left without me on board, and now it is too late to catch it. It is the train that leads to the destination of marriage and a family. I will be too old to be a father in the future."<br>• "Those kind of traumas don't go away overnight... I am walking down the street and I am afraid that immigration could come at any moment to get me... I've just leveled off a little bit mentally, and I've stopped having those dreams of imprisonment, those nightmares. But you keep thinking that immigration may come any time. You don't feel free."<br>• "Now I don't know, who am I? I lost myself. Because I'm not that person I was, that person who came to Australia. I was healthy. I was active. I had a hard time but I was happy. But now I can't laugh, I can't cry, I can't work. I can't study. I'm living in very dark place. (P02f)<br>• "I'm definitely not the person who walked into that place...I walked into that centre very healthy and I came out with four of my lower discs bulged. I'm left with permanent mental and physical disability."<br>• "We don't feel [we can] completely cope with this society because of the past. [In detention] they hurt us, they humiliated us, and they punished us. They made us very small and worse than anyone. All this sad feeling [from the past] shows us anyone could be an enemy; anyone could hurt you in the same way. We lived in fear [in detention], and still it is same thing, still I feel the same thing."	Coffey, 2010<br>Diaz, 2022<br>Witney, 2016

367

3.5 Detention Period

3.5.1 Theme 1 – Human Rights Violations in Detention.

Five subthemes were identified within theme 1:

i. **Constriction of agency and autonomy**
ii. **Endemic uncertainty**
iii. **Systemic deprivation**
iv. **Dehumanisation and perceived injustice**
v. **Loss of safety**

The curtailment of individual agency and autonomy as a result of restrictions imposed while in detention culminated in the experience of imprisonment and removal of freedom. Authorities were viewed as omnipotent in their exercise of control and curtailment of detainee agency, with a resultant feeling of futility in seeking legal advice and powerlessness in being unable to influence one's life course [78]. ASR described threatening behaviour by the authorities and the resultant loss of safety and a lack of control over their own lives, substandard living conditions, and the absence of support systems to mitigate the stressful living conditions [78].

Witney et al. (2016) noted the endemic uncertainty and the sense of living 'in limbo' whilst waiting for asylum claims to be processed, with subsequent anxiety regarding the tenuous circumstances and concerns about forced repatriation [83]. Detention was described as a prison and, at times, was perceived to be worse than prison due to the uncertainty created by an information vacuum. Increased stress levels and a reduction in sleep quality were attributed to this endemic uncertainty, with life becoming more "miserable" as the length of detention extended [78]. Paradoxically, detainees believed they would be able to manage a

longer period of detention if the exact duration were known to them [78]. Boerma et al. (2022) likewise noted that the endemic uncertainty arising from a deficiency in information and communication concerning their expectations of safety upon arrival in the Netherlands, coupled with the lack of clarity regarding detention procedures, exacerbated feelings of fear, anxiety and confusion [79]. Similar sentiments were observed in Hollis et al., [68] whereby asylum-seeking participants expressed that the inconsistent and inadequate communication they received from immigration authorities during their detention was a significant precursor to feelings of powerlessness and, consequently, a restriction of their agency.

Arshad et al. (2018) observed that the systemic deprivation characterised by neglect of fundamental needs and inadequate living conditions, including food scarcity and lack of privacy, significantly overshadowed and detracted from the pregnancy experiences of ASR women [70]. Similarly, participants in an Australian study by Passardi et al. (2022) communicated their experiences of systemic deprivation, a lack of agency, dehumanisation, exposure to violence, and a loss of safety. Dehumanisation also encompassed feeling used for political gain to ‘stop the boats’ without regard for the personal and community impact, with numerous individuals expressing regret regarding their decision to come to Australia; some wished they had perished during the maritime journey [65]. Furthermore, participants felt confronted by the lack of autonomy over their lives; deprivation was viewed as intentional, with participants feeling that guards created their own rules or had been trained to act inhumanely, which further “killed [their] minds” [65].

The distress experienced by some ASR was attributed to living a confined and highly controlled existence, with limitations governing self-determination and having no autonomy

over the most basic aspects of daily life [61, 72]. The constriction of agency and systemic deprivation was further evidenced by inadequate access to education and training, substandard sanitation, and confinement [61]. Additionally, the restrictive use of routines, limited access to finances, and the inability to engage in work or social activities beyond the limits of the centre, coupled with high levels of monitoring and security personnel, further underscored the environment of constraint [61].

Even when participants were young and healthy, systemic deprivation in neglect of basic human needs and dehumanisation experiences significantly eroded well-being [2]. Loss of safety was further conveyed by the multiple traumatic and violent experiences described, including rape or the rape of a family member, either in the host community or by a detention guard, or threats of sexual violence [65].

Loss of safety was also prominent for children, who recounted adversity and violence experienced while in detention [74]. Play therapy symbolised children's awareness of a lack of protection, even in the context of the hope that migration would increase their quality of life [74]. Similarly in Rothe et al. [76], 74 adolescents in the detention camp at Guantanamo experienced a loss of safety and psychological trauma through exposure to riots, violence, and suicide attempts made by other detainees within the camp. Adolescent girls reported the absence of privacy and limited autonomy, in conjunction with threats of sexual assault from males within the camp, as their most significant and urgent concern. Juxtaposed to this, sexual promiscuity and risk of pregnancy increased the opportunity of expulsion from detention for adolescent girls. This phenomenon was theorised to be driven by a desire to terminate the experience of detention and, hence, the limitations on agency and autonomy

[76]. Neglect of adolescents' basic needs was also evident in the unsanitary living conditions of the Guantanamo detention camp, along with a bland diet, which, in concert with the effects of heat and sensory overload, amplified tension and distress among the adolescent ASR. The adolescent participants complained of being 'treated like cattle', indicative of their experience of dehumanisation. In the chaotic conditions of detention confinement, the family unit was viewed as protective: a powerful anchor point offering safety, support and nurturance to the adolescent ASR [76].

Participants in Shishegar et al. (2021) also described a loss of autonomy and agency while in detention, attributed to feeling dehumanised and treated like criminals [66]. Furthermore, being subject to a prison-like environment was perceived as a violation and was inconsistent with expectations of safety and freedom on arrival [66]. *Johnston et al., (*2009) noted that the perceived injustice and dehumanisation associated with detention was a key precipitant of anger. For some, this was expressed outwardly, such as individual protests, as an expression of rights and agency. For others, anger was inwardly directed as an internalisation of the dominant narrative associated with being an ASR, for example, feeling 'undeserving' or that detention was a form of punishment by a higher power for previous wrongdoing, rather than associated with the quest for safety and a new life [80].

Similarly, *Diaz* et al. (2022) noted ASR experienced the enduring injustice associated with being detained in a prison-like environment, being treated like criminals, feeling 'more imprisoned than in prison', and residing in a chronic state of uncertainty endemic to the asylum process [2]. In *Mares & Jureidini* (2004), a common theme among the children was the apparent randomness of the refugee determination process and the endemic uncertainty

and anxiety that this induced, with children not being able to comprehend why some families they had met in detention had been granted visas and they had not. There was a sense of injustice arising from the arbitrary nature of this process, which, in turn, culminated in extreme feelings of anger and worthlessness [64].

3.5.2 Theme 2 – Systemic failures of healthcare provision and resultant ASR vulnerability and deprivation.

Two subthemes were identified within Theme 2:

i. **Institutional and organisational failure**
ii. **Structural barriers to medical care**

Utilising qualitative data from Immigration & Customs Enforcement (ICE) inspection reports, contracts, and detainee death reports, *Marquez* et al. (2021) determined suicide in immigration detention facilities was a by-product of institutional and organisational failure. Institutional failures, including deviations from care standards and inadequate staff training, were noted to contribute to a crisis of vulnerability and deprivation within detention systems [75]. Facilities under ICE oversight were reported as understaffed and under-resourced, failing to promptly implement federally mandated protocols for detainee well-being. This system failure was seen as central to adverse health outcomes for minority populations who are overrepresented in immigration detention in the USA, such as Latinx [75]. From 2003-2015, 150 immigrant detainees died in the custody of ICE, with 13% of deaths being due to suicide [75]. This was attributed to a failure to adequately train staff on suicide prevention and intervention, exemplifying a systemic failure that normalised substandard care that results in inequitable health outcomes for minority and marginalised detainees [75].

In a similar context, the structural barriers to accessing timely and adequate medical care articulated by Diaz et al. (2022) included delays and interruptions in medical services, insufficient access to mental health care, the denial of language interpreting services, inappropriate treatment and monitoring of COVID-19, and inadequate discharge planning. Furthermore, the majority of individuals experienced prolonged detention with restricted access to medical and mental health care, signifying a structural barrier. This situation constituted a significant impediment to addressing pre-existing health conditions and contributed to the emergence of new medical issues during the period of detention [2].

In the UK context, *Kellezi* et al. (2016) undertook a study inside Yarl's Wood Immigration Removal Centre (IRC). Of concern, 70% of the detainees reported suicidal ideation. Of those who reported having self-harmed since arrival at the IRC, 78% did not have Assessment Care in Detention and Teamwork (ACDT) plans. ACDT plans form part of a structured system implemented to monitor detainees identified as being at risk of self-harm and support vulnerable individuals, thereby ensuring they receive the necessary attention and intervention intended to avert incidents of self-harm or suicide. The absence of ACDT plans underscores a systemic failure within the detention framework that has the potential to jeopardise the well-being of vulnerable detainees [71].

Furthermore, several detainees voiced their apprehension regarding the disclosure of mental health concerns, self-harm tendencies, or suicidal ideation, fearing that such candour could adversely affect their immigration cases despite the acknowledgment that accessible mental health care constitutes a fundamental human right. Detainees reported that the experience of confinement, even for a brief duration of a few months, adversely impacted their physical

and mental health. Some detainees indicated that a formal health assessment would be timely for the identification of emerging health conditions [71].

A study conducted in the United Kingdom examining the experiences of migrant women who were detained while pregnant [70] illuminated the structural inadequacies in healthcare provision affecting an especially vulnerable population. The women in the study reported encountering significant barriers to medical care, including denial of access to timely and appropriate maternity services during detention, as well as a lack of attention from staff despite being notified of health concerns, further exemplifying systemic failures. Furthermore, the UK guidelines by the National Institute for Clinical Excellence (NICE) regarding the timely availability of antenatal care appointments [84] were not adhered to, and there was a notable absence in continuity of care. One woman had prescribed regular medication withheld, resulting in suicidality and fear for the welfare of her baby. Additionally, external medical appointments were frequently cancelled due to insufficient detention guards available to escort the women, which represents both an institutional failure and a structural barrier to obtaining medical care. Ultimately, these factors illustrate a pervasive breakdown in healthcare provision, with some women reporting the deterioration of pre-existing mental health conditions that were aggravated and neglected by the detention experience [70]. In a comparable context, Hollis (2018) records the account of a pregnant asylum seeker in immigration detention who encountered an institutional failure as a result of stringent adherence to regulations superseding fundamental human rights. This resulted in a significant loss of body weight owing to deprivation of food within her accommodation, adversely affecting her health and that of her unborn child [68].

Hollis (2018) [68] further exemplifies the structural barriers to medical care by recounting instances in which pre-existing health issues, such as chronic back pain, were minimised during detention due to insufficient pain prescriptions and a refusal to refer patients to tertiary hospitals. This systemic failure in healthcare provision rendered a participant vulnerable by compounding desolation through a clear institutional indifference to his condition. Zimmerman [69] further emphasises systemic deficiencies in healthcare provision through a case vignette involving an asylum seeker who engaged in self-harm during detention due to apprehensions regarding refoulement. The individual sustained injuries to the legs and back; however, he perceived the medical personnel as lacking in empathy, not dedicating adequate time to listen to his concerns, and similarly inadequately addressing his pain by only prescribing basic analgesics. This neglect exacerbated his vulnerability and emotional distress.

3.5.3 Theme 3 – Negative health impacts of immigration detention

Three subthemes were identified within Theme 3:

i. **Psychological health**
ii. **Physical health**
iii. **Impact on children**

The last emergent theme within the detention period, the negative health impacts of immigration detention, describes the compound downstream effect of detention. *Diaz* et al. (2022) described the psychological toll as persistent fear and insecurity, a sense of loss and despair, the emergence or worsening of anxiety and depression, and the experience of moral injury. With regard to physical health, the experience of immigration detention was observed

to result in the emergence of new physical injuries attributable to violence, the onset of new medical conditions (such as obesity and hyperlipidaemia), as well as difficulties in the management of pre-existing chronic medical conditions (for instance, HIV/AIDS), and the implications of COVID-19 infection and its sequelae [2]. Notably, all participants were young and healthy, with few pre-migration medical conditions. Yet, the experience of detention culminated in poor physical and psychological health [2].

In the UK context, most detainees reported high rates of anxiety, distress and depression [71]. The 'mental distress' described by ASR in *Campbell and Steel* (2014) was noted to encompass broad feelings of depression, anguish, frustration, stress, hopelessness and the loss of dignity, with all seven ASR participants experiencing these symptoms. Participants reported a significant increase in these symptoms while residing in detention centres, connecting the environment of immigration detention with mental distress. Health and social service professionals providing services to asylum-seekers described the mental distress experienced by asylum-seekers in the immigration detention environment, noting that policy conditions that restrict movement in detention were linked to the experience of suffering and psychological ill-health for ASR [61]. Mounting hopelessness and demoralisation were a dominant and recurring theme in *Coffey* et al. (2010), with all participants highlighting their struggle to maintain hope and becoming increasingly demoralised while detained. For the majority, this was a gradual and insidious deterioration coupled with mounting fatigue and loss of vitality. This, in conjunction with a reduction of the ability to act with a sense of purpose, was viewed as a precursor to poor psychological health [3].

Similarly, the nine participants in Boerma et al. (2022) expressed that the hopelessness of immigration detention, coupled with increased rumination in the absence of meaningful stimulation, evoked memories of past traumas experienced in their country of origin, resulting in a retriggering of trauma and psychological distress; six of these individuals reported experiencing suicidal thoughts during their detention, while three reported having attempted suicide [79]. Isolation implemented in detention settings is perceived as a catalyst that engenders a feedback loop characterised by heightened uncertainty, criminalisation, disappointment, and a pervasive sense of futility in pursuing protection. This process further exacerbates issues such as re-traumatization alongside the cumulative effects of increased anxiety, depression, symptoms of post-traumatic stress disorder (PTSD), and suicidal tendencies [79].

Kronick et al. (2015) aimed to understand the experiences of detained children and families who sought asylum in Canada, with 20 families participating in the study. Families reported a range of symptoms indicating the deterioration of psychological well-being as expressed by detained infants, children and adolescents, with children – more so in those under six years – frequently displaying externalising oppositional and aggressive behaviours. Furthermore, reduced appetite or food intake occurred in the majority of interviewed children, with two infants requiring more frequent nursing and increased soothing. Significant anxiety (including separation anxiety), selective mutism, mood and post-traumatic stress symptoms were also reported, indicative of substantial psychological burden, postulated to be a correlate of the detention experience [82].

In *Mares & Jureidini*, (2004), very high levels of psychopathology were found in both child and adult asylum seekers, primarily being attributed to traumatic experiences in detention and, for the children, the impact of indefinite detention on their caregivers. All children had at least one parent with a psychiatric illness. Of the 10 children aged 6-17 years, all met criteria for both post-traumatic stress disorder (PTSD) and major depressive disorder, with suicidal ideation. Eight children (80%), including three pre-adolescents, had made significant self-harm attempts, and seven (70%) had symptoms of an anxiety disorder. Half reported persistent severe somatic symptoms such as headaches and abdominal pain. The majority (80%) of preschool-age children were identified with developmental delay or emotional disturbance [81].

In Arsenijevic et al. (2018), participants reported compromised psychological well-being and psychosomatic symptoms resulting from prolonged and excessive stress compounded by cumulative trauma, which included the detention experience. This phenomenon stemmed from a confluence of experiences associated with pre-migration, migration, and their subsequent immigration detention experience. Symptoms included headaches, tremors, and heart palpitations, with some individuals engaging in maladaptive behaviours such as substance abuse and self-harm. Conversely, others resorted to altruism, rationalising their circumstances and seeking solace in religious faith and familial connections [77]. In Rothe (2003), the impact of cumulative trauma was observed in the interplay among pre-existing trauma, the maritime crossing, and the experiences associated with detention. Notably, 94% of the boys and 96% of the girls were classified in the highest category of traumatic stress symptoms. The reported behavioural indicators included frequent crying (49% males; 63% females), aggressive behaviour (42% males; 55% females), nightmares (74% males; 96%

females), appetite disturbances (51% males; 48% females), sleep disturbances (85% males; 96% females), enuresis (45% males; 48% females), and encopresis (8% males; 11% females). These findings highlighted a significant deterioration in psychological well-being [76].

3.6 Post-detention period

3.6.1 Theme 4 – Changes in self-construct, relationships and worldview.

Two subthemes were identified within Theme 4:

i. **Fragmentation of identity and changes in self-perception**

ii. **Erosion of trust and social connectivity.**

In Witney et al. (2016), narrative disruption was measured as a subjective schema that orders details of an individual's past, present and future within a meaningful construct of the self. The indefinite nature of detention and the associated trauma were perceived to fragment the individuals' concept of their narrative and self-construct. The isolation of detention centres and the limited interaction with the external environment, frequent relocations among detention facilities, and prolonged periods in solitary confinement served as significant factors in the deterioration of trust and social connectivity. Furthermore, the gradual decline in self-confidence and self-regulation stemming from solitary confinement within detention centres exemplified a reduction in agency, leading to adverse alterations in self-perception and a consequent decrease in social connectivity [83].

This is contrasted by some participants in Passardi et al. (2022), who experienced a change in self-construct through feeling haunted by overwhelming questions of guilt and personal culpability and whether individual shortcomings may explain the reason for their detention experience [65]. The prevalent negative perception of the world and self was manifested

through a decline in trust towards others, coupled with an inability to experience interconnectedness or to establish, nurture, and sustain meaningful friendships. This deterioration of trust and social connectivity was further exacerbated for certain participants who reported having observed acts of self-harm among their peers, including instances of suicide attempts, which frequently occurred both during and after their detention. These experiences often correlated with episodes of sexual assault experienced in detention. The erosion of trust in interpersonal relationships and the incapacity to form close bonds were considered 'damaging' and perceived as detrimental to their self-concept, reinforcing a negative worldview and self-image [65].

The disempowerment of ASR due to the experience of detention contributed to a diminished sense of self and identity, thereby fragmenting the self-perception that ASR had of themselves as autonomous and competent individuals with agency over their lives [77]. In *Cleveland (2018),* immigration detention was viewed as a form of symbolic violence through the removal of individual agency and choice, including the liberty to organise one's daily life, as fundamental to human identity, self-perception and autonomy [72]. Detention, therefore, initiated the process of disempowerment by subjecting ASR to measures that heralded, for an indeterminate length of time, the removal of basic human rights and freedoms, which can be viewed as precursors to the change of fragmentation of identity and self-construct [72].

Almost all participants in *Coffey et al. (2010)* who were previously detained underwent significant transformations in their perceptions of self and identity and a loss of agency and sociability due to the disabling effect of their psychological state in the aftermath of detention. Fractured relationships, social withdrawal and personality change, were seen as a

direct consequence of detention – most prominently, increased irritability and impatience, distrust of others and increased self-doubt, all of which were accompanied by increased rumination [3]. A dampened sense of agency was prominent, as evidenced by impaired capacity for initiative and goal-directed activity, including activities of daily living, language and skill acquisition, and sustaining study or employment, which were all viewed as overwhelming [3].

3.6.2 Theme 5 – Enduring impact on mental and physical health

Two subthemes were identified within Theme 5:

i. **Continued psychological sequelae**
ii. **Physical health deterioration**

In *Passardi* et al. (2022), all but one participant raised ongoing psychological sequelae in the aftermath of their detention experience. Depression, emotional numbness, sleeping disturbance, obsessive-compulsive behaviours, irritability, and ongoing sadness associated with their experiences in detention were all reported. The impact on physical health included emergent physical conditions or the exacerbation of pre-existing physical conditions, such as skin diseases, renal and bladder calculi, alopecia, ear and eye problems and Alzheimer's disease [65]. In the post-detention phase, the enduring impact of the detention experience was evidenced by continued psychological sequelae, with participants experiencing a profound sense of injustice and being aggrieved by the combined experiences of deprivation, lack of agency, violence and dehumanisation encountered after arrival in Australia. The Australian government was viewed as culpable in rendering them feeling irreparably

damaged. Additionally, participants felt hopeless due to having lost productive years of their lives in detention owing to a process viewed as meaningless [65].

Following their detention, all participants in Coffey et al, (2010) reported persistent psychological sequelae due to the ongoing insecurity and prolonged uncertainty regarding their futures and those of their families stemming from the constraints imposed by their visa conditions. These restrictions, coupled with the loss of productive working years while detained, were perceived as acts of injustice affecting all participants post-release. Moreover, participants expressed profound grievance regarding the intrinsic nature of the detention experience, unable to be rationalised and integrated with their suffering appearing entirely devoid of meaning. As a result, depression, demoralisation, and disturbances in concentration and memory, along with persistent anxiety, were frequently reported. The lasting nature of these detrimental psychological effects was found to persist for several years following their release [3].

Immigration detention exerts significant psychological stress and trauma on children, who exhibit responses characterised by extreme distress, fear, and impaired functioning [82]. Detention was observed to be detrimental to most children after an average duration of 56.4 days. The symptoms incurred did not resolve immediately, leading to sustained adverse psychiatric sequelae well after their release from detention. This phenomenon is partially indicative of cumulative exposure to trauma [74, 82].

4. Discussion

We report here the key themes emerging from the systematic synthesis of 20 qualitative studies associated with the physical and mental health impact of immigration detention on ASR children and adults. The themes could be divided into those relating to the detention period and those consequent to detention.

Immigration detention has been associated with significant negative impacts on the physical and mental health of ASR adults and children [18, 19, 25, 85, 86], ASR are at increased risk of developing mental disorders due to the traumatic experiences that served as push factors in seeking asylum [87, 88]. The confluence of pre-existing trauma compounded by the migration and post-migration journey further influences the overall mental health trajectory with high levels of psychiatric morbidity arising from detention experiences [18, 48, 49, 51, 52, 57, 85, 89]. In detention, the often inadequate conditions and inhumane treatment endured, encompassing family separation, exposure to violence, discrimination, deprivation of human rights, prolonged confinement, isolation devoid of distractions, overcrowding, unsanitary conditions, and substandard food quality, likely catalyse the detrimental health effects associated with immigration detention [2].

Furthermore, the conditions of detention are often aggravated by the mental distress of being an unauthorised arrival, by the threat of deportation or repatriation, including the threat of violence and ongoing persecution [8, 90]. These systemic barriers and institutional failings culminate in the creation of vulnerabilities and a decline in mental health and increased

suicidal ideation [2]. Suicide was the leading cause of premature death for those in immigration detention in Australia [91].

The apparatus and function of immigration detention may be seen as a strategy to deter or remove asylum seekers from the destination country [92]. The literature suggested that a deterrence model predominates, whereby detention centres are used as a mechanism to deter others from seeking protection in industrialised countries, as well as to encourage those who do arrive, to return to their country of origin [93]. It is not uncommon for detainees to relinquish their right to appeal their detention as their emotional, financial and health resources become depleted during the prolonged wait time and the associated endemic uncertainty [94]. These factors of protracted uncertainty and reduced self-agency contribute to an environment within detention where human rights violations become possible.

Detention is characterised as a prison-like setting, featuring limited space, imposing security fences, and the presence of guards, all of which disrupt and inhibit detainees' capacity for self-determination and engagement in meaningful activities [61, 66, 78]. The freedom to determine one's daily routine is significantly restricted as a direct consequence of prevailing detention policy conditions [61, 65]. High levels of stress have been reported, originating from fear of deportation to the country of origin and a lack of trust in the host communities inhabited by ASR, as a direct consequence of immigration policy [95]. Far-reaching effects of immigration detention further include a decline in healthcare usage [96, 97], distrust of authorities, and wariness in engaging with institutions [98, 99]. When navigating the healthcare system, the risk of being re-detained or deported at the hands of medical providers

may take precedence over seeking medical care [75]. It, therefore, has been argued that ASR view healthcare as a means of “biopolitical surveillance” [75].

The significant psychological toll of detention is underpinned by a current of arbitrariness and uncertainty, with a pervasive sense of injustice and moral injury serving as a potent mechanism for traumatic stress [2]. The resultant feeling of powerlessness was strongly correlated with all types of mental health symptoms [72]. Other experiences, including indeterminacy over the length of detention, long periods of inactivity, boredom, fear of deportation, family concerns, loneliness, and perceived injustice of detention, are also associated with high levels of mental health symptoms [72]. ASR who were buoyed by the hope of safety, freedom and a better life and the ability to actively engage in the realisation of these hopes were buffered against mental distress. Conversely, a sense of powerlessness as a consequence of the immigration detention experience was most strongly correlated with PTSD, depression and anxiety symptoms, with detention sometimes triggering re-traumatisation in trauma survivors [72]. Detention, even for brief periods in relatively adequate conditions, was detrimental to ASR mental health [18].

Across the included studies, the notion of protracted and endemic uncertainty as a result of the immigration detention experience and beyond was evident. Waiting is inherent to the asylum-seeking process and increases uncertainty, which in turn has a harmful effect on well-being [100]. Waiting for an indeterminate period for the outcome of one’s refugee status determination generates feelings of powerlessness and serves to enact power relations through the constant reminder of subordination to a dominant ‘other’s’ arbitrary power [72] [101]. The consequent loss of agency has been found to diminish the ability to engage socially

[102]. Persistent feelings of low self-esteem and shame have also been associated with the inhibition of goal-directed activities, thereby reducing productivity and engagement with the external world [103]. Furthermore, there is a correlation between asylum seekers living in a 'chronic state of uncertainty' [15] and psychological and somatoform disorders [100], with prolonged detention and protracted uncertainty regarding the future, maintaining anxiety, depression, hopelessness and mental distress [104, 105].

4.1 Children and Families

The United Nations Convention on the Rights of the Child (UNCRC) stipulates that the detention of children should be a "measure of last resort" (United Nations Office of the High Commissioner for Human Rights [UNHCR], 1989, Article 37. B [106]). Despite this, children who arrive as refugees or asylum seekers in over 60 countries may be subject to immigration detention [37].

Children in detention are particularly vulnerable and are known to experience developmental delays, emotional trauma, and long-term psychological effects that can impact their development [107]. Detention can be viewed as a form of toxic stress with the potential to interfere with children's capacity to recover from previous trauma and psychopathology [74]. Mares & Jureidini (2004) noted that mental health and overall functioning deteriorated as immigration detention became protracted [81]. The pervasive deterioration raises ethical and moral concerns for clinicians bearing witness to a phenomenon that is at odds with international research demonstrating sustained improvement over time among resettled ASR and post-conflict populations [108]. Interactions with mental health clinicians were noted to be humanising and psychologically safe for adolescents, with this hypothesised as serving an

environment fostering psychological containment and regulation [76]. Notably, in cases where immigration law took precedence over state health and child protection jurisdictions, clinicians and staff felt morally duty-bound yet immobilised by their inability to effect significant change for children and their parents with severe psychiatric illness and distress [81]. It is important to note that even brief detention appears to be acutely distressing for children, and that constraining and threatening detention environments may serve as an acute stressor, evoking helplessness and fear [73].

4.2 Physical Health

While somatic illness is acknowledged as salient within populations affected by trauma, a limited number of studies have explored somatic conditions potentially linked to detention in ASR [18, 109]. Furthermore, such conditions are frequently conflated with physical health indices, which exhibit considerable overlap and interrelation. Disentangling somatic illness in ASR from physical and mental health indices will be important in understanding the impacts of detention on physical health.

4.3 Key Stakeholders–- Clinicians and support staff

The staff-detainee relationship was noted to be of paramount importance in de-escalating potential occurrences of self-harm and suicide and addressing mental health concerns. However, detention staff indicated that they could not appropriately recognise and deal with severe mental health distress owing to their lack of training or experience [51, 71]. Similarly, volunteer health professionals cited that detention staff were not able to recognise that mental health could deteriorate both in detention as well as in pregnancy and thus, detained pregnant women were not believed when they expressed concerns about their deteriorating

mental health. Consequently, appropriate referrals for women to support their mental health were not made [70]. The argument was forwarded that immigration detention facilities are inadequately staffed and resourced, resulting in a systemic failure to implement competently and promptly mandated protocols pertaining to ASR well-being [75].

4.4 Strengths and limitations

This qualitative synthesis represents, to the authors' knowledge, the first endeavour to synthesise existing qualitative literature concerning the physical and mental health implications of immigration detention on asylum seekers and refugees (ASR). A thorough review of qualitative evidence was conducted to incorporate previously overlooked data, thereby offering a more comprehensive understanding of ASR experiences and the health consequences associated with immigration detention. Preliminary thematic frameworks were discussed iteratively within the research team to agree on the emergent themes. A limitation is the complex nature of synthesising studies employing diverse qualitative methodologies; nonetheless, despite this limitation, coherent and integrated findings and themes were successfully extracted, providing unique and significant insights.

The majority of studies concentrate on sample populations situated within the post-detention context rather than on individuals who are detained at the time of recruitment. This observation highlights the possibility of bias in their retrospective accounts of physical and mental health sequelae. This review underscores the dearth of comprehensive physical health data pertaining to asylum seekers and refugees who have experienced immigration detention. We acknowledge that all authors of this systematic review are healthcare researchers and/or clinicians with a vested interest in the health of refugees and asylum

seekers. While we do not consider our dedication to advancing healthcare for a marginalised and occasionally politicised group as a limitation of our work, we do recognise that our professional and personal perspectives inevitably influence our interpretation and critique of the literature.

4.5 Clinical Implications and Future Research

There exists a necessity for further research aimed at developing and assessing interventions to assist asylum seekers and refugees (ASR) in managing the significant changes in their lives subsequent to the experience of immigration detention, with a particular emphasis on their physical and mental health. The results of this synthesis may prove beneficial for health professionals and policymakers engaged in immigration detention, as they may assist in informing the future development of treatment protocols tailored to the needs of ASR patients. The findings provide future detention policy review with insight into the inherent and ameliorable harms and risks encountered by ASRs, both during and following the period of detainment.

Conclusions

This systematic review and thematic synthesis of 20 studies underscores the significant long-term effects that immigration detention imposes on the psychological well-being of asylum seekers and refugees, both during their period of detention and subsequently. The cumulative consequences of detention highlight the necessity for comprehensive support and healthcare services and associated clinicians and support staff that are tailored to the needs of individuals who have been detained. The findings provide a synthesis of the current literature, illustrating the persistent psychological health implications linked to immigration detention. In contrast, observations regarding physical health have been infrequently

documented throughout the studies. This underscores the necessity for host nations to adopt practical and ethical policies aimed at alleviating these adverse effects, even in the context of addressing other policy objectives.

Data availability

Data has been provided as part of the submitted article.

Acknowledgments

NIL

Author contributions

BS, DH, LMA and SS reviewed the studies for inclusion. BS analysed the data, and BS, DH, SR, and SS interpreted the data. BS drafted the manuscript and prepared all figures and tables. All authors reviewed and approved the final manuscript.

Funding

Nil

Declaration of interest

The authors declare no conflict of interest.

Supplementary material

Supplementary File 1:

Search Strategy

1 asylum seek*.mp. [mp=title, book title, abstract, original title, name of substance word, subject heading word, floating sub-heading word, keyword heading word, organism supplementary concept word, protocol supplementary concept word, rare disease supplementary concept word, unique identifier, synonyms, population supplementary concept word, anatomy supplementary concept word]

2 asylum-seek*.mp. [mp=title, book title, abstract, original title, name of substance word, subject heading word, floating sub-heading word, keyword heading word, organism supplementary concept word, protocol supplementary concept word, rare disease supplementary concept word, unique identifier, synonyms, population supplementary concept word, anatomy supplementary concept word]

3 (asylum seeker* or asylum-seeker*).mp. [mp=title, book title, abstract, original title, name of substance word, subject heading word, floating sub-heading word, keyword heading word, organism supplementary concept word, protocol supplementary concept word, rare disease supplementary concept word, unique identifier, synonyms, population supplementary concept word, anatomy supplementary concept word]

4 asylum claim*.mp. [mp=title, book title, abstract, original title, name of substance word, subject heading word, floating sub-heading word, keyword heading word, organism supplementary concept word, protocol supplementary concept word, rare disease supplementary concept word, unique identifier, synonyms, population supplementary concept word, anatomy supplementary concept word]

5 (refugee* or migrant* or immigrant*).mp. [mp=title, book title, abstract, original title, name of substance word, subject heading word, floating sub-heading word, keyword heading word, organism supplementary concept word, protocol supplementary concept word, rare disease supplementary concept word, unique identifier, synonyms, population supplementary concept word, anatomy supplementary concept word]

6 refugees.mp. [mp=title, book title, abstract, original title, name of substance word, subject heading word, floating sub-heading word, keyword heading word, organism supplementary concept word, protocol supplementary concept word, rare disease supplementary concept word, unique identifier, synonyms, population supplementary concept word, anatomy supplementary concept word]

7 1 or 2 or 3 or 4 or 5 or 6

8 (detention* or detention or immigration detention*).mp. [mp=title, book title, abstract, original title, name of substance word, subject heading word, floating sub-heading word, keyword heading word, organism supplementary concept word, protocol supplementary concept word, rare disease supplementary concept word, unique identifier, synonyms, population supplementary concept word, anatomy supplementary concept word]

9 (detain or detained).mp. [mp=title, book title, abstract, original title, name of substance word, subject heading word, floating sub-heading word, keyword heading word, organism supplementary concept word, protocol supplementary concept word, rare disease supplementary concept word, unique identifier, synonyms, population supplementary concept word, anatomy supplementary concept word]

10 (imprison* or imprisoned*).mp. [mp=title, book title, abstract, original title, name of substance word, subject heading word, floating sub-heading word, keyword heading word, organism supplementary concept word, protocol supplementary concept word, rare disease

supplementary concept word, unique identifier, synonyms, population supplementary concept word, anatomy supplementary concept word]

11 incarcerate*.mp. [mp=title, book title, abstract, original title, name of substance word, subject heading word, floating sub-heading word, keyword heading word, organism supplementary concept word, protocol supplementary concept word, rare disease supplementary concept word, unique identifier, synonyms, population supplementary concept word, anatomy supplementary concept word]

12 reception cent*.mp. [mp=title, book title, abstract, original title, name of substance word, subject heading word, floating sub-heading word, keyword heading word, organism supplementary concept word, protocol supplementary concept word, rare disease supplementary concept word, unique identifier, synonyms, population supplementary concept word, anatomy supplementary concept word]

13 temporary protection.mp. [mp=title, book title, abstract, original title, name of substance word, subject heading word, floating sub-heading word, keyword heading word, organism supplementary concept word, protocol supplementary concept word, rare disease supplementary concept word, unique identifier, synonyms, population supplementary concept word, anatomy supplementary concept word]

14 custody*.mp. [mp=title, book title, abstract, original title, name of substance word, subject heading word, floating sub-heading word, keyword heading word, organism supplementary concept word, protocol supplementary concept word, rare disease supplementary concept word, unique identifier, synonyms, population supplementary concept word, anatomy supplementary concept word]

15 (prison or jail*).mp. [mp=title, book title, abstract, original title, name of substance word, subject heading word, floating sub-heading word, keyword heading word, organism

supplementary concept word, protocol supplementary concept word, rare disease supplementary concept word, unique identifier, synonyms, population supplementary concept word, anatomy supplementary concept word]

16 8 or 9 or 10 or 11 or 12 or 13 or 14 or 15

17 (health* or health outcomes* or mental health* or physical health* or quality of life*).mp. [mp=title, book title, abstract, original title, name of substance word, subject heading word, floating sub-heading word, keyword heading word, organism supplementary concept word, protocol supplementary concept word, rare disease supplementary concept word, unique identifier, synonyms, population supplementary concept word, anatomy supplementary concept word]

18 7 and 16 and 17

19 (qualitative* or mixed-methods* or narrative*).mp. [mp=title, book title, abstract, original title, name of substance word, subject heading word, floating sub-heading word, keyword heading word, organism supplementary concept word, protocol supplementary concept word, rare disease supplementary concept word, unique identifier, synonyms, population supplementary concept word, anatomy supplementary concept word]

20 18 and 19

Search Strategy Customised for databases

History | Save | Delete | Print view | Export | Email | Combine | using And / Or | Collapse

#18	#6 AND #15 AND #16 AND [english]/lim	812
#17	#6 AND #15 AND #16	839
#16	'health'	7,650,660
#15	#7 OR #8 OR #9 OR #10 OR #11 OR #12 OR #13 OR #14	49,791
#14	'correctional facility'	17,602
#13	'custodial care'	7,599
#12	temporary AND protection	2,929
#11	'asylum seeker center'	45
#10	'prisoner'	20,204
#9	imprison* OR imprisoned*	3,407
#8	detain OR detained	2,348
#7	detention	5,904
#6	#1 OR #2 OR #3 OR #4 OR #5	61,372
#5	'immigrant'	29,475
#4	'migrant'	22,893
#3	'refugee'	16,339
#2	asylum AND claim	58
#1	asylum AND seeker	1,435

Supplementary File 2: Quality Assessment Utilising the Joanna Briggs Institute (JBI) Checklist for Qualitative Research

Study	Q1. S there congruity between the stated philosophical perspective and the research methodology?	Q2. Is there congruity between the research methodology and the research question or objectives?	Q3. Is there congruity between the research methodology and the methods used to collect data?	Q4. Is there congruity between the research methodology and the representation and analysis of data?	Q5. Is there congruity between the research methodology and the interpretation of results?	Q6. Is there a statement locating the researcher culturally or theoretically?	Q7. Is the influence of the researcher on the research, and vice-versa, addressed?	Q8. Are participants, and their voices, adequately represented?	Q9. Is the research ethical according to current criteria or, for recent studies, and is there evidence of ethical approval by an appropriate body?	Q10. Do the conclusions drawn in the research report flow from the analysis, or interpretation, of the data?
Arsenijevic et al. 2018	UNCLEAR	YES	YES	YES	YES	NO	NO	YES	YES	YES
Arshad et al. 2018	UNCLEAR	YES	YES	YES	YES	NO	NO	YES	YES	YES
Boerma et al, 2022	UNCLEAR	YES	YES	YES	YES	NO	NO	YES	N/A	YES
Campbell et al. 2013	YES	YES	YES	YES	YES	NO	NO	YES	YES	YES
Cleveland et al. 2018	UNCLEAR	YES	YES	YES	YES	NO	NO	YES	YES	YES
Coffey et al., 2010	UNCLEAR	YES	YES	YES	YES	NO	NO	YES	YES	YES
Diaz et al. 2022	UNCLEAR	YES	YES	YES	YES	NO	NO	YES	YES	YES
Hollis, 2018	YES	YES	YES	YES	YES	YES	NO	YES	YES	YES

Johnston et al., 2009	YES	YES	YES	YES	YES	YES	NO	YES	YES	YES
Kellezi et al. 2016	UNCLEAR	UNCLEAR	UNCLEAR	UNCLEAR	YES	NO	NO	YES	NO	YES
Kronick et al. 2015	YES	YES	YES	YES	YES	NO	NO	YES	YES	YES
Kronick et al. 2018	YES	YES	YES	YES	YES	NO	YES	YES	YES	YES
Mares & Jureidini, 2004	UNCLEAR	YES	YES	UNCLEAR	YES	NO	NO	YES	YES	YES
Marquez et al. 2021	YES	YES	YES	YES	YES	NO	NO	N/A	N/A	NO
Passardi et al. 2022	YES	YES	YES	YES	YES	YES	NO	YES	YES	YES
Puthoopparambil et al. 2015	UNCLEAR	YES	YES	YES	YES	NO	NO	YES	YES	YES
Rothe et al., 2003	UNCLEAR	UNCLEAR	UNCLEAR	UNCLEAR	YES	NO	NO	YES	NO	YES
Shishehgar et al. 2021	UNCLEAR	YES	YES	YES	YES	YES	YES	YES	YES	YES
Witney and Bates 2016	YES	YES	YES	YES	YES	NO	NO	YES	NO	YES
Zimmerman et al., 2012	YES	YES	YES	YES	YES	NO	No	Yes	Yes	Yes

References

1. Sharples R, Briskman L. Introducing the Politics of Deterrence, Detainment and Dehumanisation. United Kingdom: Emerald Publishing Limited; 2024.
2. Diaz C, Ortiz V, Sanchez L, Fernandez J, Andrade EA, Akiyama MJ, et al. Harmful by Design—a Qualitative Study of the Health Impacts of Immigration Detention. Journal of general internal medicine : JGIM. 2023;38(9):2030-7. doi: 10.1007/s11606-022-07914-6.
3. Coffey GJ, Kaplan I, Sampson RC, Tucci MM. The meaning and mental health consequences of long-term immigration detention for people seeking asylum. Social Science & Medicine. 2010;70(12):2070-9. doi: 10.1016/j.socscimed.2010.02.042.
4. Steel Z, Liddell BJ, Bateman-Steel CR, Zwi AB. Global protection and the health impact of migration interception. PLoS Med. 2011;8(6):e1001038-e. doi: 10.1371/journal.pmed.1001038.
5. Fekete L. The deportation machine: Europe, asylum and human rights. Race & class. 2005;47(1):64-78. doi: 10.1177/0306396805055083.
6. Hernández D, Eason JM, Goldsmith PR, Abel RD, McNeely A, Miller HV, et al. With Mass Deportation Comes Mass Punishment: Punitive Capacity, Health, and Standards in U.S. Immigrant Detention. Routledge Handbooks. 1 ed. ABINGDON: Routledge; 2018. p. 260-9.
7. Morey BN. Mechanisms by which anti-immigrant stigma exacerbates racial/ethnic health disparities. American journal of public health (1971). 2018;108(4):460-3. doi: 10.2105/AJPH.2017.304266.
8. Alexander BC. Detention of Asylum-seekers in the United States. Human Rights Brief 7. 2000:20-1.
9. Keller AS, Ford D, Sachs E, Ba, Rosenfeld B, Trinh-Shevrin C, et al. The Impact of Detention on the Health of Asylum Seekers. 2003. p. 383.
10. Wood LCN. Impact of punitive immigration policies, parent-child separation and child detention on the mental health and development of children. BMJ paediatrics open. 2018;2(1):e000338-e. doi: 10.1136/bmjpo-2018-000338.
11. Franco K, Patkler, Caitlin, Reiter, Keramet Punishing status and the punishment status quo: Solitary confinement in US Immigration detention facilities, 2013- 2017. Punishment & Society. 2020. doi: https://doi.org/10.31235/osf.io/zdy7f.
12. Crock M. Refugees and Rights: Taylor & Francis; 2017.
13. Wallace RMM, Janeczko FAW, Islam R, Bhuiyan JH, Bhuiyan JH, Islam MR, et al. The Concept of Asylum in International Law. United States: BRILL; 2013. p. 133-58.
14. Laban CJ, Gernaat HBPE, Komproe IH, van der Tweel I, De Jong JTVM. Postmigration Living Problems and Common Psychiatric Disorders in Iraqi Asylum Seekers in the Netherlands. The journal of nervous and mental disease. 2005;193(12):825-32. doi: 10.1097/01.nmd.0000188977.44657.1d.
15. Afifi TD, Afifi WA, Acevedo Callejas M, Shahnazi A, White A, Nimah N. The Functionality of Communal Coping in Chronic Uncertainty Environments: The Context of Palestinian Refugees in Lebanon. Health communication. 2019;34(13):1585-96. doi: 10.1080/10410236.2018.1514682.
16. Ahmad F, Othman N, Hynie M, Bayoumi AM, Oda A, McKenzie K. Depression-level symptoms among Syrian refugees: findings from a Canadian longitudinal study. Journal of mental health (Abingdon, England). 2021;30(2):246-54. doi: 10.1080/09638237.2020.1765998.

17. Blackmore R, Boyle JA, Fazel M, Ranasinha S, Gray KM, Fitzgerald G, et al. The prevalence of mental illness in refugees and asylum seekers: A systematic review and meta-analysis. PLoS medicine. 2020;17(9):e1003337-e. doi: 10.1371/journal.pmed.1003337.
18. Robjant K, Hassan R, Katona C. Mental health implications of detaining asylum seekers: systematic review. British journal of psychiatry. 2009;194(4):306-12. doi: 10.1192/bjp.bp.108.053223.
19. Robjant K, Robbins I, Senior V. Psychological distress amongst immigration detainees: A cross-sectional questionnaire study. British journal of clinical psychology. 2009;48(3):275-86. doi: 10.1348/014466508X397007.
20. Steel Z, Chey T, Silove D, Marnane C, Bryant RA, van Ommeren M. Association of Torture and Other Potentially Traumatic Events With Mental Health Outcomes Among Populations Exposed to Mass Conflict and Displacement: A Systematic Review and Meta-analysis. JAMA : the journal of the American Medical Association. 2009;302(5):537-49. doi: 10.1001/jama.2009.1132.
21. Steel Z, Liddell BJ, Bateman-Steel CR, Zwi AB. Global protection and the health impact of migration interception. PLoS medicine. 2011;8(6):e1001038-e. doi: 10.1371/journal.pmed.1001038.
22. Hedrick K, Armstrong G, Coffey G, Borschmann R. Self-harm in the Australian asylum seeker population: A national records-based study. Elsevier BV; 2019.
23. Fazel M, Silove D. Detention Of Refugees: Australia Has Given Up Mandatory Detention Because It Damages Detainees' Mental Health. BMJ (Online). 2006;332(7536):251-2. doi: 10.1136/bmj.332.7536.251.
24. Hollings J, Samuilova M, Petrova-Benedict R. Health, migration and border management: analysis and capacity-building at Europe’s borders. International Journal of Public Health. 2012;57(2):363-9. doi: 10.1007/s00038-011-0319-8.
25. Steel Z, Momartin S, Silove D, Coello M, Aroche J, Tay KW. Two year psychosocial and mental health outcomes for refugees subjected to restrictive or supportive immigration policies. Social Science & Medicine. 2011;72(7):1149-56. doi: 10.1016/j.socscimed.2011.02.007.
26. A Report By Physicians for Human Rights and the Bellevue/NYU Program for Survivors of Torture. From persecution to prison: The health consequences of detention for asylum seekers. 2003:219.
27. Venters H, Dasch-Goldberg D, Rasmussen A, Keller AS. Into the Abyss: Mortality and Morbidity among Detained Immigrants. Human rights quarterly. 2009;31(2):474-95. doi: 10.1353/hrq.0.0074.
28. Hassan G, Kirmayer, LJ, Mekki-, Berrada A. Q, C., el Chammay, R., Deville-Stoetzel, , J.B. Y, A., Jefee-Bahloul, H., Barkeel-Oteo, A., , Coutts A, Song, S. & Ventevogel, P. . Culture, Context and the Mental Health and Psychosocial Wellbeing of Syrians: A Review for Mental Health and Psychosocial Support staff working with Syrians Affected by Armed Conflict. UNHCR. 2015.
29. Klein A, Williams L. Immigration Detention in the Community: Research on the Experiences of Migrants Released from Detention Centres in the UK. Population space and place. 2012;18(6):741-53. doi: 10.1002/psp.1725.
30. Silverman SJ, Massa E. Why Immigration Detention is Unique. Population space and place. 2012;18(6):677-86. doi: 10.1002/psp.1720.

31. Li SSY, Liddell BJ, Nickerson A. The Relationship Between Post-Migration Stress and Psychological Disorders in Refugees and Asylum Seekers. Current psychiatry reports. 2016;18(9):82. doi: 10.1007/s11920-016-0723-0.

32. Bronstein I, Montgomery P. Psychological Distress in Refugee Children: A Systematic Review. Clinical child and family psychology review. 2011;14(1):44-56. doi: 10.1007/s10567-010-0081-0.

33. Amarasena L, Samir N, Sealy L, Hu N, Rostami MR, Isaacs D, et al. Offshore detention: cross-sectional analysis of the health of children and young people seeking asylum in Australia. Arch Dis Child. 2023;108(3):185-91. doi: 10.1136/archdischild-2022-324442.

34. Zwi K, Mares S, Nathanson D, Tay AK, Silove D. The impact of detention on the social–emotional wellbeing of children seeking asylum: a comparison with community-based children. European child & adolescent psychiatry. 2018;27(4):411-22. doi: 10.1007/s00787-017-1082-z.

35. Mares S. The Mental Health of Children and Parents Detained on Christmas Island: Secondary Analysis of an Australian Human Rights Commission Data Set. Health Hum Rights. 2016;18(2):219-32.

36. Mares S. Fifteen years of detaining children who seek asylum in Australia – evidence and consequences. Australas Psychiatry. 2016;24(1):11-4. doi: 10.1177/1039856215620029.

37. Fazel M, Karunakara U, Newnham EA. Detention, denial, and death: migration hazards for refugee children. The Lancet global health. 2014;2(6):e313-e4. doi: 10.1016/S2214-109X(14)70225-6.

38. Dudley M, Steel Z, Mares S, Newman L. Children and young people in immigration detention. Current opinion in psychiatry. 2012;25(4):285-92. doi: 10.1097/YCO.0b013e3283548676.

39. Newman LKFP, Steel ZM. The Child Asylum Seeker: Psychological and Developmental Impact of Immigration Detention. Child and adolescent psychiatric clinics of North America. 2008;17(3):665-83. doi: 10.1016/j.chc.2008.02.009.

40. Fazel MD, Reed RVM, Panter-Brick CP, Stein AP. Mental health of displaced and refugee children resettled in high-income countries: risk and protective factors. The Lancet (British edition). 2012;379(9812):266-82. doi: 10.1016/S0140-6736(11)60051-2.

41. Heptinstall E, Sethna V, Taylor E. PTSD and depression in refugee children: Associations with pre-migration trauma and post-migration stress. European child & adolescent psychiatry. 2004;13(6):373-80. doi: 10.1007/s00787-004-0422-y.

42. Amarasena L, Samir N, Sealy L, Hu N, Rostami MR, Isaacs D, et al. Offshore detention: cross-sectional analysis of the health of children and young people seeking asylum in Australia. Archives of disease in childhood. 2023;108(3):185-91. doi: 10.1136/archdischild-2022-324442.

43. von Werthern M, Grigorakis G, Vizard E. The mental health and wellbeing of Unaccompanied Refugee Minors (URMs). Child abuse & neglect. 2019;98:104146. doi: 10.1016/j.chiabu.2019.104146.

44. Mares S. Mental health consequences of detaining children and families who seek asylum: a scoping review. European child & adolescent psychiatry. 2021;30(10):1615-39. doi: 10.1007/s00787-020-01629-x.

45. Filges T, Montgomery E, Kastrup M. The Impact of Detention on the Health of Asylum Seekers: A Systematic Review. Research on social work practice. 2018;28(4):399-414. doi: 10.1177/1049731516630384.

46. Hanes G, Chee J, Mutch R, Cherian S. Paediatric asylum seekers in Western Australia: Identification of adversity and complex needs through comprehensive refugee health assessment. Wiley; 2019. p. 1367.

47. Filges T, Bengtsen E, Montgomery E, Kildemoes MW. The impact of detention on the health of asylum seekers: An updated systematic review: A systematic review. Campbell systematic review. 2024;20(3):e1420-n/a. doi: 10.1002/cl2.1420.

48. Filges T, Montgomery E, Kastrup M. The Impact of Detention on the Health of Asylum Seekers. Research on social work practice. 2018;28(4):399-414. doi: 10.1177/1049731516630384.

49. Verhülsdonk I, Shahab M, Molendijk M. Prevalence of psychiatric disorders among refugees and migrants in immigration detention: systematic review with meta-analysis. BJPsych Open. 2021;7(6):e204. Epub 2021/11/15. doi: 10.1192/bjo.2021.1026.

50. Filges T, Montgomery E, Kastrup M, Jørgensen AMK. The Impact of Detention on the Health of Asylum Seekers: A Systematic Review. Campbell systematic review. 2015;11(1):1-104. doi: 10.4073/csr.2015.13.

51. Bosworth M. "Mental Health in Immigration Detention: A Literature Review". Review into the Welfare in Detention of Vulnerable Persons. Criminal Justice, Borders and Citizenship Research Paper No 2732892. 2016:37. doi: Available at SSRN: https://ssrn.com/abstract=2732892.

52. Storm T, Engberg M. The impact of immigration detention on the mental health of torture survivors is poorly documented--a systematic review. Dan Med J. 2013;60(11):A4728. PubMed PMID: 24192244.

53. Van Hout M-C, Lungu-Byrne C, Germain J. Migrant health situation when detained in European immigration detention centres: a synthesis of extant qualitative literature. International journal of prisoner health. 2020;16(3):221-36. doi: 10.1108/IJPH-12-2019-0074.

54. Wembri E CM, McClunie-Trust P. A report on the impact of immigration detention on the health and well-being of refugees & asylum seekers: A meta-ethnography. Ethnographic Edge. 2024;7:21-41. doi: doi: 10.24135/ee.v7i1.267.

55. Institute JB. JBI Critical Appraisal Checklist for Qualitative Research. Adelaide: Joanna Briggs Institute. 2017.

56. O'Brien BC, Harris IB, Beckman TJ, Reed DA, Cook DA. Standards for Reporting Qualitative Research: A Synthesis of Recommendations. Academic medicine. 2014;89(9):1245-51. doi: 10.1097/ACM.0000000000000388.

57. Von Werthern M, Robjant K, Chui Z, Schon R, Ottisova L, Mason C, et al. The impact of immigration detention on mental health: a systematic review. Springer Science and Business Media LLC; 2018.

58. K. H. Chapter 4: Critical appraisal of qualitative research. In: Noyes J, Booth A, Hannes K, Harden A, Harris J, Lewin S, et al., editors. Supplementary Guidance for Inclusion of Qualitative Research in Cochrane Systematic Reviews of Interventions [Internet]. Version 1 (updated August 2011). Cochrane Collaboration Qualitative Methods Group. 2011

59. Lucas PJ, Baird J, Arai L, Law C, Roberts HM. Worked examples of alternative methods for the synthesis of qualitative and quantitative research in systematic reviews. BMC medical research methodology. 2007;7(1):4-. doi: 10.1186/1471-2288-7-4.

60. Barnett-Page E, Thomas J. Methods for the synthesis of qualitative research: a critical review. BMC medical research methodology. 2009;9(1):59-. doi: 10.1186/1471-2288-9-59.

61. Campbell EJ, Steel EJ. Mental distress and human rights of asylum seekers. Journal of public mental health. 2015;14(2):43-55. doi: 10.1108/JPMH-06-2013-0040.

62. Coffey GJ, Kaplan I, Sampson RC, Tucci MM. The meaning and mental health consequences of long-term immigration detention for people seeking asylum. Social science & medicine (1982). 2010;70(12):2070-9. doi: 10.1016/j.socscimed.2010.02.042.

63. Johnston V, Allotey P, Mulholland K, Markovic M. Measuring the health impact of human rights violations related to Australian asylum policies and practices: A mixed methods study. BMC international health and human rights. 2009;9(1):1-. doi: 10.1186/1472-698X-9-1.

64. Mares S, Jureidini J. Psychiatric assessment of children and families in immigration detention – clinical, administrative and ethical issues. Australian and New Zealand journal of public health. 2004;28(6):520-6. doi: 10.1111/j.1467-842X.2004.tb00041.x.

65. Passardi S, Hocking DC, Morina N, Sundram S, Alisic E. Moral injury related to immigration detention on Nauru: a qualitative study. European journal of psychotraumatology. 2022;13(1):2029042. doi: 10.1080/20008198.2022.2029042.

66. Shishehgar S, Gholizadeh L, DiGiacomo M, Davidson PM. A qualitative study of experiences of asylum-seeker women of living in detention centres: confinement versus safety. Contemporary nurse : a journal for the Australian nursing profession. 2021;57(1-2):51-64. doi: 10.1080/10376178.2021.1927771.

67. Witney A, Bates G. Narrative integration of identity following trauma Life-stories of immigrants granted asylum in Australia following prolonged detention. Narrative Inquiry. 2016;26:88-107. doi: 10.1075/ni.26.1.05wit.

68. Hollis J. The psychosocial experience of UK immigration detention. International journal of migration, health and social care. 2019;15(1):76-89. doi: 10.1108/IJMHSC-04-2018-0024.

69. Zimmerman SE, Chatty D, Nørredam ML. Health needs and access to care in immigration detention: perceptions of former detainees. International journal of migration, health and social care. 2012;8(4):180-5. doi: 10.1108/17479891211287094.

70. Arshad F, Haith-Cooper M, Palloti P. The experiences of pregnant migrant women in detention: A qualitative study. British journal of midwifery. 2018;26(9):591-6. doi: 10.12968/bjom.2018.26.9.591.

71. Kellezi B BM. Mental health, suicidal thoughts and self-harm inside immigration detention. SSRN Electron J. 2016. doi: doi: 10.2139/ssrn.2867358.

72. Cleveland J, Kronick R, Gros H, Rousseau C. Symbolic violence and disempowerment as factors in the adverse impact of immigration detention on adult asylum seekers' mental health. International journal of public health. 2018;63(8):1001-8. doi: 10.1007/s00038-018-1121-7.

73. Kronick R, Rousseau C, Cleveland J. Asylum-Seeking Children's Experiences of Detention in Canada: A Qualitative Study. American journal of orthopsychiatry. 2015;85(3):287-94. doi: 10.1037/ort0000061.

74. Kronick R, Rousseau C, Cleveland J. Refugee children's sandplay narratives in immigration detention in Canada. European child & adolescent psychiatry. 2018;27(4):423-37. doi: 10.1007/s00787-017-1012-0.

75. Marquez BA, Marquez-Velarde G, Eason JM, Aldana L. Pushing them to the edge: Suicide in immigrant detention centers as a product of organizational failure. Social science & medicine (1982). 2021;283:114177. doi: 10.1016/j.socscimed.2021.114177.

76. Rothe EM, Castillo-Matos H, Busquets R. Posttraumatic stress symptoms in Cuban adolescent refugees during camp confinement. Adolescent psychiatry. 2003;27:97-124.

77. Arsenijević J, Burtscher D, Ponthieu A, Severy N, Contenta A, Moissaing S, et al. “I feel like I am less than other people”: Health-related vulnerabilities of male migrants travelling alone on their journey to Europe. Social science & medicine (1982). 2018;209:86-94. doi: 10.1016/j.socscimed.2018.05.038.

78. Puthoopparambil SJ, Ahlberg BM, Bjerneld M. “A prison with extra flavours”: experiences of immigrants in Swedish immigration detention centres. International journal of migration, health and social care. 2015;11(2):73-85. doi: 10.1108/IJMHSC-10-2014-0042.

79. Boerma RS, van G-O, II, Smid GE. ['They're still adding more pain on me': psychological effects of immigration detention]. Tijdschr Psychiatr. 2022;64(3):139-44. PubMed PMID: 35420142.

80. Johnston V, Allotey P, Mulholland K, Markovic M. Measuring the health impact of human rights violations related to Australian asylum policies and practices: a mixed methods study. BMC International Health and Human Rights. 2009;9(1):1. doi: 10.1186/1472-698X-9-1.

81. Mares S, Jureidini J. Psychiatric assessment of children and families in immigration detention--clinical, administrative and ethical issues. Aust N Z J Public Health. 2004;28(6):520-6. doi: 10.1111/j.1467-842x.2004.tb00041.x. PubMed PMID: 15707200.

82. Kronick R, Rousseau C, Cleveland J, McLeigh JD, Spaulding W. Asylum-Seeking Children's Experiences of Detention in Canada: A Qualitative Study. American journal of orthopsychiatry. 2015;85(3):287-94. doi: 10.1037/ort0000061.

83. Witney A BG. Narrative integration of identity following trauma: Life-stories of immigrants granted asylum in Australia following prolonged detention. . Narrat Inquiry 2016;26:88-107. doi: doi: 10.1075/ni.26.1.05wit.

84. National Institute for Health and Care Excellence. Antenatal care for uncomplicated pregnancies [NG201]. NICE. 2021:63.

85. Newman L. Seeking Asylum-Trauma, Mental Health, and Human Rights: An Australian Perspective. Journal of trauma & dissociation. 2013;14(2):213-23. doi: 10.1080/15299732.2013.724342.

86. Silove D, Austin P, Steel Z. No Refuge from Terror: The Impact of Detention on the Mental Health of Trauma-affected Refugees Seeking Asylum in Australia. Transcultural psychiatry. 2007;44(3):359-93. doi: 10.1177/1363461507081637.

87. Silove D, Sinnerbrink I, Field A, Manicavasagar V, Steel Z. Anxiety, depression and PTSD in asylum-seekers: Associations with pre-migration trauma and post-migration stressors. British journal of psychiatry. 1997;170(3):351-7. doi: 10.1192/bjp.170.4.351.

88. Fazel M, Wheeler J, Danesh J. Prevalence of serious mental disorder in 7000 refugees resettled in western countries: a systematic review. The Lancet (British edition). 2005;365(9467):1309-14. doi: 10.1016/S0140-6736(05)61027-6.

89. Silove D. The Psychosocial Effects of Torture, Mass Human Rights Violations, and Refugee Trauma: Toward an Integrated Conceptual Framework. The journal of nervous and mental disease. 1999;187(4):200-7. doi: 10.1097/00005053-199904000-00002.

90. Keller AS, Rosenfeld B, Trinh-Shevrin C, Meserve C, Sachs E, Leviss JA, et al. Mental health of detained asylum seekers. Lancet. 2003;362(9397):1721-3. doi: 10.1016/S0140-6736(03)14846-5.

91. Procter NG, Leo D, Newman L. Suicide and self-harm prevention for people in immigration detention. Medical journal of Australia. 2013;199(11):730-2. doi: 10.5694/mja13.10804.

92. Díaz J. Immigration Policy, Criminalization and the Growth of the Immigration Industrial Complex: Restriction, Expulsion, and Eradication of the Undocumented in the U.S. 1. Criminology, criminal justice, law & society. 2011;12(2):35.
93. Silove D, Steel Z, Watters C. Policies of Deterrence and the Mental Health of Asylum Seekers. JAMA. 2000;284(5):604-11. doi: 10.1001/jama.284.5.604.
94. R M. Death in detention: Medical and mental health consequences of indefinite detention of immigrants in the United States. . Seattle Journal for Social Justice. 2008;7(2):693-736.
95. Hacker K, Chu J, Leung C, Marra R, Pirie A, Brahimi M, et al. The impact of Immigration and Customs Enforcement on immigrant health: Perceptions of immigrants in Everett, Massachusetts, USA. Social Science & Medicine. 2011;73(4):586-94. doi: 10.1016/j.socscimed.2011.06.007.
96. Rhodes SD, Mann L, Simán FM, Song E, Alonzo J, Downs M, et al. The impact of local immigration enforcement policies on the health of immigrant Hispanics/Latinos in the United States. American journal of public health (1971). 2015;105(2):329-37. doi: 10.2105/AJPH.2014.302218.
97. Watson T. Inside the Refrigerator: Immigration Enforcement and Chilling Effects in Medicaid Participation. American economic journal Economic policy. 2014;6(3):313-38. doi: 10.1257/pol.6.3.313.
98. Pedraza FI, Nichols VC, LeBrón AMW. Cautious Citizenship: The Deterring Effect of Immigration Issue Salience on Health Care Use and Bureaucratic Interactions among Latino US Citizens. Journal of health politics, policy and law. 2017;42(5):925-60. doi: 10.1215/03616878-3940486.
99. Nichols VC, LeBrón AMW, Pedraza FI. Policing Us Sick: The Health of Latinos in an Era of Heightened Deportations and Racialized Policing. PS, political science & politics. 2018;51(2):293-7. doi: 10.1017/S1049096517002384.
100. Phillimore J, Cheung SY. The violence of uncertainty: Empirical evidence on how asylum waiting time undermines refugee health. Social science & medicine (1982). 2021;282:114154. doi: 10.1016/j.socscimed.2021.114154.
101. Turnbull S. 'Stuck in the middle': Waiting and uncertainty in immigration detention. Time & society. 2016;25(1):61-79. doi: 10.1177/0961463X15604518.
102. Herman JL, Leader E. Trauma and recovery: the aftermath of violence - from domestic abuse to political terror. 1994. p. 76-7.
103. Brandstätter V, Bernecker K. Persistence and disengagement in personal goal pursuit. Annual Review of Psychology. 2022;73(1):271-99.
104. Steel Z, Momartin S, Bateman C, Hafshejani A, Silove DM, Everson N, et al. Psychiatric status of asylum seeker families held for a protracted period in a remote detention centre in Australia. Australian and New Zealand journal of public health. 2004;28(6):527-36. doi: 10.1111/j.1467-842X.2004.tb00042.x.
105. Sultan A, O'Sullivan K. Psychological disturbances in asylum seekers held in long term detention: a participant–observer account. Med J Aust. 2001;175(11-12):593-6. doi: 10.5694/j.1326-5377.2001.tb143740.x.
106. Assembly UNG, Directorate CHR. Convention on the Rights of the Child: Human Rights Directorate; 1991.
107. Zwi K, Mares S, Nathanson D, Tay AK, Silove D. The impact of detention on the social–emotional wellbeing of children seeking asylum: a comparison with community-based children. European child & adolescent psychiatry. 2018;27:411-22.

108. Mollica RF, Sarajlić N, Chernoff M, Lavelle J, Vuković IS, Massagli MP. Longitudinal Study of Psychiatric Symptoms, Disability, Mortality, and Emigration Among Bosnian Refugees. JAMA : the journal of the American Medical Association. 2001;286(5):546-54. doi: 10.1001/jama.286.5.546.

109. Knights F, Munir S, Ahmed H, Hargreaves S. Initial health assessments for newly arrived migrants, refugees, and asylum seekers. BMJ (Online). 2022;377:e068821-e. doi: 10.1136/bmj-2021-068821.

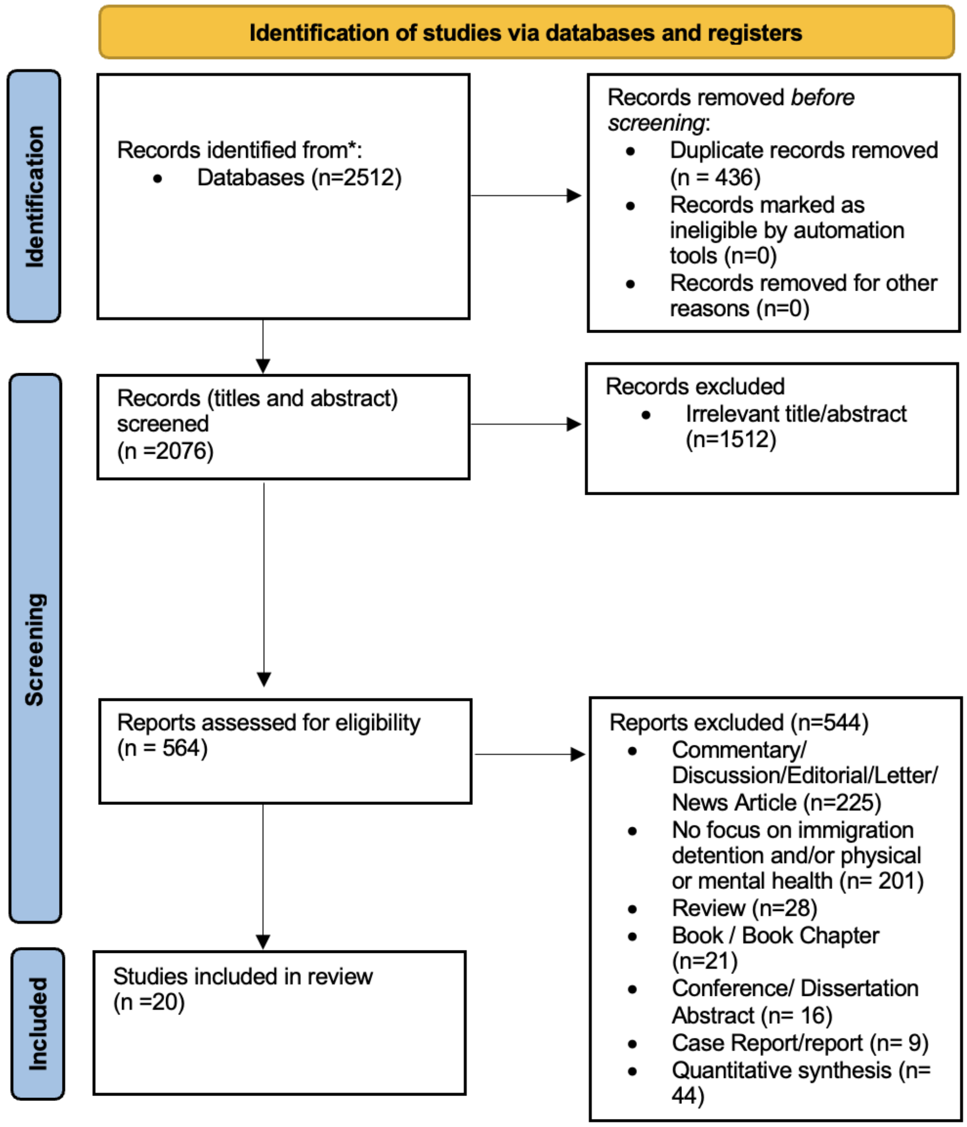

Fig.1 PRISMA Flow diagram for the systematic review of qualitative studies

Figure 1 Click here to access/download;Figure;Figure 2.docx

Theme 1 – Human Rights Violations in Detention.

i. Constriction of agency and autonomy
ii. Endemic uncertainty
iii. Systemic deprivation
iv. Dehumanisation and perceived injustice
v. Loss of safety

Theme 2 – Systemic failures of healthcare provision and resultant ASR vulnerability and deprivation.

i. Institutional and organisational failure
ii. Structural barriers to medical care

Theme 3 – Negative health impacts of immigration detention

i. Psychological health
ii. Physical health
iii. Impact on children

Theme 4 – Changes in self-construct, relationships and worldview.

- Fragmentation of identity and changes in self-perception
- Erosion of trust and social connectivity.

Theme 5 – Enduring impact on mental and physical health

- Continued psychological sequelae
- Physical health deterioration

Figure 2 –Thematic Qualitative Synthesis

Click here to access/download
Supporting Information
PRISMA_2020_checklist_FINAL.docx

MONASH University

16 February 2025

Response to Reviewers – Manuscript Number PGPH-D-25-00362

3/01/2025

Dear Dr. Sherif,

Thank you for submitting your manuscript for review by PLOS Global Public Health. After careful consideration, we have decided that your manuscript does not meet our standards for systematic and scoping reviews. We are, therefore, rejecting your manuscript.

PLOS Global Public Health considers systematic reviews and meta-analyses for publication; however, these submissions must address a clear and defined research question, conduct a systematic and comprehensive literature review, and use clearly reported, reproducible, and systematic methods to identify, select, and critically appraise relevant research. Our author guidelines for systematic reviews are at https://journals.plos.org/globalpublichealth/s/submission-guidelines#loc-systematic-reviews-and-meta-analyses.

In the case of your submission, we note that language restrictions are imposed on the literature searches without adequate justification. There is increasing recognition that the exclusion of studies published in other languages can increase the risk of bias in systematic reviews. We feel that any restrictions should be justified based on scientific reasons rather than resource constraints. As a result, we have concerns about the comprehensiveness of the review and the validity of the conclusions.

As a result of these concerns, we cannot consider the manuscript for publication at PLOS Global Public Health. at this time. However, if you are able to address the concerns discussed above, we

Department of Psychiatry
Faculty of Medicine, Nursing and Health Sciences, School of Clinical Sciences at Monash Health
Monash Medical Centre, Level 3, P Block, 246 Clayton Road, Clayton VIC 3168 Australia
T: +61 3 9594 1479 F: +61 3 9594 6499
E: suresh.sundram@monash.edu
www.monash.edu
ABN 12 377 614 012 CRICOS Provider 00008C

may be able to consider a revised version of the manuscript. This would however require a substantial amount of work and thus, would be considered as a new submission. If you are able to revise the manuscript to respond to the concerns noted above, please refer to the original submission in the covering letter and accompany the submission by a rebuttal letter that responds to the comments made on the original manuscript.

I am sorry that we cannot be more positive on this occasion, but I hope you understand the reasons for this decision.

Dear Dr. Dhond and colleagues,

We express our sincere gratitude to you and the reviewers for the insightful feedback provided regarding our manuscript. We have meticulously reviewed the comments and have implemented revisions to address the concerns raised.

The editorial office instructed us that papers published in languages other than English must be included in any systematic review. Consequently, we conducted a thorough search strategy that encompassed non-English language papers, resulting in the identification of seven relevant studies. Among these, one paper fulfilled the inclusion criteria and has been integrated into our manuscript for your re-evaluation.

This endeavour has enabled us to adequately address the concerns pertaining to the comprehensiveness of the review and the validity of the conclusions drawn.

Department of Psychiatry
Faculty of Medicine, Nursing and Health Sciences, School of Clinical Sciences at Monash Health
Monash Medical Centre, Level 3, P Block, 246 Clayton Road, Clayton VIC 3168 Australia
T: +61 3 9594 1479 F: +61 3 9594 6499
E: suresh.sundram@monash.edu
www.monash.edu
ABN 12 377 614 012 CRICOS Provider 00008C

We have attached the revised manuscript for your perusal. We appreciate your consideration of this manuscript.

Yours sincerely,

Bafreen Sherif

B.Pharm (Hons), PgDipClinPharm (Distinc), MScClinPharm (Hons), RegPharm AU/NZ

Monash University, Dept of Psychiatry, School of Clinical Sciences, Faculty of Medicine, Nursing and Health Sciences, Monash University, Victoria, Australia

Department of Psychiatry
Faculty of Medicine, Nursing and Health Sciences, School of Clinical Sciences at Monash Health
Monash Medical Centre, Level 3, P Block, 246 Clayton Road, Clayton VIC 3168 Australia
T: +61 3 9594 1479 F: +61 3 9594 6499
E: suresh.sundram@monash.edu
www.monash.edu
ABN 12 377 614 012 CRICOS Provider 00008C
