# Supplementary material for: A systematic review of qualitative research on the physical and mental health impacts of immigration detention on asylum seekers and refugees
Source: PLOS Glob Public Health. 2025 Oct 29;5(10):e0005196. doi: 10.1371/journal.pgph.0005196 (PMC12571329; doi:10.1371/journal.pgph.0005196)
Supplement: Attachment — Submitted filename: Response to Reviewers- PLOS Global Public Health.docx [file pgph.0005196.s008.docx]

12 February 2025

**Response to Reviewers – Manuscript Number PGPH-D-25-00362**

3/01/2025

Dear Dr. Sherif,

Thank you for submitting your manuscript for review by PLOS Global Public Health. After careful consideration, we have decided that your manuscript does not meet our standards for systematic and scoping reviews. We are, therefore, rejecting your manuscript.

PLOS Global Public Health considers systematic reviews and meta-analyses for publication; however, these submissions must address a clear and defined research question, conduct a systematic and comprehensive literature review, and use clearly reported, reproducible, and systematic methods to identify, select, and critically appraise relevant research. Our author guidelines for systematic reviews are at <https://journals.plos.org/globalpublichealth/s/submission-guidelines#loc-systematic-reviews-and-meta-analyses>.

In the case of your submission, we note that language restrictions are imposed on the literature searches without adequate justification. There is increasing recognition that the exclusion of studies published in other languages can increase the risk of bias in systematic reviews. We feel that any restrictions should be justified based on scientific reasons rather than resource constraints. As a result, we have concerns about the comprehensiveness of the review and the validity of the conclusions.

As a result of these concerns, we cannot consider the manuscript for publication at PLOS Global Public Health. at this time. However, if you are able to address the concerns discussed above, we may be able to consider a revised version of the manuscript. This would however require a substantial amount of work and thus, would be considered as a new submission. If you are able to revise the manuscript to respond to the concerns noted above, please refer to the original submission in the covering letter and accompany the submission by a rebuttal letter that responds to the comments made on the original manuscript.

I am sorry that we cannot be more positive on this occasion, but I hope you understand the reasons for this decision.

Dear Dr. Dhond and colleagues,

We express our sincere gratitude to you and the reviewers for the insightful feedback provided regarding our manuscript. We have meticulously reviewed the comments and have implemented revisions to address the concerns raised.

The editorial office instructed us that papers published in languages other than English must be included in any systematic review. Consequently, we conducted a thorough search strategy that encompassed non-English language papers, resulting in the identification of seven relevant studies. Among these, one paper fulfilled the inclusion criteria and has been integrated into our manuscript for your re-evaluation.

This endeavour has enabled us to adequately address the concerns pertaining to the comprehensiveness of the review and the validity of the conclusions drawn.

We have attached the revised manuscript for your perusal. We appreciate your consideration of this manuscript.

Yours sincerely,

Bafreen Sherif

B.Pharm (Hons), PgDipClinPharm (Distinc), MScClinPharm (Hons), RegPharm AU/NZ

*Monash University, Dept of Psychiatry, School of Clinical Sciences, Faculty of Medicine, Nursing and Health Sciences, Monash University, Victoria, Australia*
