# Supplementary material for: A systematic review of qualitative research on the physical and mental health impacts of immigration detention on asylum seekers and refugees
Source: PLOS Glob Public Health. 2025 Oct 29;5(10):e0005196. doi: 10.1371/journal.pgph.0005196 (PMC12571329; doi:10.1371/journal.pgph.0005196)
Supplement: Attachment — Submitted filename: PLOS Global Public Health Review_NS2025.odt [file pgph.0005196.s006.odt]

PLOS Global Public Health Review: A systematic review of qualitative research on the physical and mental health impacts of immigration detention on asylum seekers and refugees.
I would like to thank the authors for choosing to shed light on the plight of such a population (asylum seekers and immigrants) who are not receiving the attention needed due to the many compounding factors, including the changes in the global context where a population's needs are easily disregarded or deprioritised.
Definitions:
The topic of immigration encompasses health, legal issues, protection, and general social support. This implies that the definitions provided in the articles should conform to standards established by sources that address immigration broadly. Utilising the UNHCR, the International Organisation for Migration, or any relevant sources will enhance the clarity of both the reader's understanding and the article itself, given the multidisciplinary nature of the topic. 
The three main terms to clarify in this article are immigrants, asylum seekers, and refugees. It is important for refugees that they cannot move until a green light is granted in some countries; asylum seekers can be at the border or already in the country. Your article focuses on people already in the detention centres, hence the need to clarify this.
Lines 140 and 141 attempt to define asylum seeking; please see the comments above.
Lines 755, 756, and 757 attempt to define a detention centre, which is already useful. My question would be whether this should be on a separate annexe or included in the introduction. It will be important to have these definitions covered in this manuscript.
Length of the process:
The article attempts to clarify how long the asylum-seeking process might take but does not address refugee status. How long does it take to get your results when you are seeking asylum? How long does it take to obtain refugee status?
Clarity:
On lines 98, 99 and 100, the sentence will need to be adjusted with a consideration for another word apart from it (assuming you are referring to the manuscript).
On lines 120, 121 and 122, is it possible to use another word or term that aligns with asylum seeking and being a refugee? The word recruitment sounds like people are happy being in one of the two categories.
Line 62 mentions only two reviewers, under general methods, please include all study authors supporting the literature selection, data extraction and doing the review.
Lines 553 impact on children: 
While this is information from the studies, it is important to help the reader understand what you are trying to explain. Why are children not falling under the same category for psychological health and physical health? 
Lines 712 – 719 talk about children. I am wondering why you are not putting this information together.
Line 588: Protection is mentioned here, and it is the only time I have not read anything in the paper on protection. Reading your themes, a way to bring back protection, legal, health, and social support would highlight the need for a multidisciplinary approach to this topic.
Flow chart: It should include all the databases searched and the grey literature.
Results: They do not mention anything linked to physical health, as explained under line 165, but the mental health of adults and children. I would have a similar remark for the conclusion, too.
