# Supplementary material for: A systematic review of qualitative research on the physical and mental health impacts of immigration detention on asylum seekers and refugees
Source: PLOS Glob Public Health. 2025 Oct 29;5(10):e0005196. doi: 10.1371/journal.pgph.0005196 (PMC12571329; doi:10.1371/journal.pgph.0005196)
Supplement: Attachment — Submitted filename: Rebuttal Letter 27_08_25 FINAL.odt [file pgph.0005196.s009.odt]

PGPH-D-25-00362
A systematic review of qualitative research on the physical and mental health impacts of immigration detention on asylum seekers and refugees
PLOS Global Public Health

Dear Dr. Sherif,

Thank you for submitting your manuscript to PLOS Global Public Health. After careful consideration, we feel that it has merit but does not fully meet PLOS Global Public Health’s publication criteria as it currently stands. Therefore, we invite you to submit a revised version of the manuscript that addresses the points raised during the review process. 

Please submit your revised manuscript by Aug 31 2025 11:59PM. If you will need more time than this to complete your revisions, please reply to this message or contact the journal office at globalpubhealth@plos.org. When you're ready to submit your revision, log on tohttps://www.editorialmanager.com/pgph/ and select the 'Submissions Needing Revision' folder to locate your manuscript file.

Please include the following items when submitting your revised manuscript:
A rebuttal letter that responds to each point raised by the editor and reviewer(s). You should upload this letter as a separate file labelled 'Response to Reviewers'.
A marked-up copy of your manuscript that highlights changes made to the original version. You should upload this as a separate file labelled 'Revised Manuscript with Track Changes'.
An unmarked version of your revised paper without tracked changes. You should upload this as a separate file labelled 'Manuscript'.
Guidelines for resubmitting your figure files are available below the reviewer comments at the end of this letter.

We look forward to receiving your revised manuscript.

Kind regards,

Claire J Standley
Academic Editor
PLOS Global Public Health

Journal Requirements:
If the reviewer comments include a recommendation to cite specific previously published works, please review and evaluate these publications to determine whether they are relevant and should be cited. There is no requirement to cite these works unless the editor has indicated otherwise. 

Please review your reference list to ensure that it is complete and correct. If you have cited papers that have been retracted, please include the rationale for doing so in the manuscript text, or remove these references and replace them with relevant current references. Any changes to the reference list should be mentioned in the rebuttal letter that accompanies your revised manuscript. If you need to cite a retracted article, indicate the article’s retracted status in the References list and also include a citation and full reference for the retraction notice.

Additional Editor Comments (if provided):

In addition to the comments provided through peer review, please consider the additional suggestions and comments provided in the attached file. In particular, consider where text can be streamlined (especially when similar information is already provided in tables/figures), and recommend focusing the Discussion more squarely on where the qualitative data inform and expand on what was already known from quantitative systematic reviews.

Authors’ Response: We thank the editor for these helpful suggestions, which have guided us in refining the manuscript. The Introduction, Methods, and Discussion have been revised to improve clarity, concision, and focus. Please refer to the manuscript for further clarification.


 [Note: HTML markup is below. Please do not edit.]

Reviewers' comments:

Reviewer's Responses to Questions

Comments to the Author

1. Does this manuscript meet PLOS Global Public Health’s publication criteria? Is the manuscript technically sound, and do the data support the conclusions? The manuscript must describe methodologically and ethically rigorous research with conclusions that are appropriately drawn based on the data presented.
Reviewer #1: Partly

2. Has the statistical analysis been performed appropriately and rigorously?
Reviewer #1: N/A

3. Have the authors made all data underlying the findings in their manuscript fully available (please refer to the Data Availability Statement at the start of the manuscript PDF file)?

The PLOS Data policy requires authors to make all data underlying the findings described in their manuscript fully available without restriction, with rare exception. The data should be provided as part of the manuscript or its supporting information, or deposited to a public repository. For example, in addition to summary statistics, the data points behind means, medians and variance measures should be available. If there are restrictions on publicly sharing data—e.g. participant privacy or use of data from a third party—those must be specified.
Reviewer #1: Yes

4. Is the manuscript presented in an intelligible fashion and written in standard English?

PLOS Global Public Health does not copyedit accepted manuscripts, so the language in submitted articles must be clear, correct, and unambiguous. Any typographical or grammatical errors should be corrected at revision, so please note any specific errors here.
Reviewer #1: Yes

5. Review Comments to the Author

Please use the space provided to explain your answers to the questions above. You may also include additional comments for the author, including concerns about dual publication, research ethics, or publication ethics. (Please upload your review as an attachment if it exceeds 20,000 characters)
Reviewer #1: PLOS Global Public Health Review: A systematic review of qualitative research on the physical and mental health impacts of immigration detention on asylum seekers and refugees.

I would like to thank the authors for choosing to shed light on the plight of such a population (asylum seekers and immigrants) who are not receiving the attention needed due to the many compounding factors, including the changes in the global context where a population's needs are easily disregarded or deprioritised.

Definitions:
The topic of immigration encompasses health, legal issues, protection, and general social support. This implies that the definitions provided in the articles should conform to standards established by sources that address immigration broadly. Utilising the UNHCR, the International Organisation for Migration, or any relevant sources will enhance the clarity of both the reader's understanding and the article itself, given the multidisciplinary nature of the topic.


The three main terms to clarify in this article are immigrants, asylum seekers, and refugees. It is important for refugees that they cannot move until a green light is granted in some countries; asylum seekers can be at the border or already in the country. Your article focuses on people already in the detention centres, hence the need to clarify this.

 Lines 140 and 141 attempt to define asylum seeking; please see the comments above.

Authors’ Response: Thank you for this useful suggestion. We have now included the following text at lines 160 – 201” 

“In the immigration context, detention centres are facilities used to confine individuals—typically asylum seekers, refugees, or irregular migrants—pending the outcome of their immigration case or removal from the host country. They are widely characterised as prison-like environments, often with limited personal space, high-security fencing, and constant surveillance by guards, which restrict autonomy and opportunities for meaningful activity [12-14]. Daily routines are heavily regulated by policy [12, 15], and the environment is often associated with a fear of deportation, distrust of host communities, and the generation of persistent stress [16]. Broader effects include reduced healthcare usage [17, 18], mistrust of authorities, and wariness in engaging with institutions [19, 20]. In some cases, fear of re-detention or deportation during healthcare interactions has led detainees to view health services as a form of “biopolitical surveillance” [21]. 
Given the variation in detention systems across countries and the differing terminology associated with detainees in various studies, this review draws on definitions from the United Nations High Commissioner for Refugees (UNHCR) and the International Organisation for Migration (IOM) to clarify key terms.  According to the UNHCR, a refugee is someone who, “owing to a well-founded fear of being persecuted for reasons of race, religion, nationality, membership of a particular social group or political opinion, is outside their country of nationality and is unable or unwilling to avail themselves of the protection of that country,” as recognised under the 1951 Convention Relating to the Status of Refugees and its 1967 Protocol. Refugee status is conferred following a formal determination process. Although it is a contravention of the Convention Relating to the Status of Refugees for signatory nations to detain refugees, refugees from other nations may still be detained.  An asylum seeker is someone who has applied for recognition as a refugee and is awaiting a decision on their claim. Asylum seekers may present themselves at a border or already be within the territory of the receiving country [22]. They are entitled to remain in the country of application until a decision is made regarding their application. 

This review focuses specifically on asylum seekers and refugees (ASR) who are held in immigration detention facilities, whether at borders, offshore sites, or within the territory of the host country. We use the collective term “ASR” throughout to reflect both the legal distinctions and the frequent interchangeability of these terms in academic and policy discourse, while acknowledging the lack of consistent usage across the literature.”


 Lines 755, 756, and 757 attempt to define a detention centre, which is already useful. My question would be whether this should be on a separate annexe or included in the introduction. It will be important to have these definitions covered in this manuscript.

Authors’ Response: Thank you. We have now included the following text at lines 160-167: 

“In the immigration context, detention centres are facilities used to confine individuals—typically asylum seekers, refugees, or irregular migrants—pending the outcome of their immigration case or removal from the host country. They are widely characterised as prison-like environments, often with limited personal space, high-security fencing, and constant surveillance by guards, which restrict autonomy and opportunities for meaningful activity [12-14]. Daily routines are heavily regulated by policy [12, 15], and the environment is often associated with a fear of deportation, distrust of host communities, and the generation of persistent stress [16].”


Length of the process:
The article attempts to clarify how long the asylum-seeking process might take, but does not address refugee status. How long does it take to get your results when you are seeking asylum? How long does it take to obtain refugee status?

Authors’ Response: It is acknowledged that a subtheme (theme 1 ii) of uncertainty and protracted waiting emerged from the data. However, our systematic review did not aim to investigate the function of time on the impact of immigration detention, nor the refugee determination process. 

Clarity:
On lines 98, 99 and 100, the sentence will need to be adjusted with a consideration for another word apart from it (assuming you are referring to the manuscript).

Authors’ Response: Amended: In accordance with PRISMA, SRQR, and JBI guidelines, we implemented a rigorous and transparent methodology to facilitate replication of the systematic review process and strengthen the reliability of our results.

On lines 120, 121 and 122, is it possible to use another word or term that aligns with asylum seeking and being a refugee? The word recruitment sounds like people are happy being in one of the two categories.

Authors’ Response: Sentence amended: “The predominant body of research has focused on forced migrant populations in the post-detention context, rather than recruiting participants during their detention period.”

Line 62 mentions only two reviewers. Under general methods, please include all study authors supporting the literature selection, data extraction and doing the review. 

Authors’ Response: To address this point, L225 (under general Methods section, 2.1) has been amended to: “All authors (BS, DH, LA, SS, SR) contributed to the systematic review process. Title and abstract screening were performed independently by two authors (BS and DH), with disagreements resolved through consensus with additional authors (LMA, SR and SS). Data extraction was carried out independently by two reviewers (BS and DH) and then cross-checked by the remaining team (LMA, SS, SR). BS performed the data analysis, and data interpretation was undertaken collaboratively by BS, DH, SS, and SR.”  

Additionally, Line 62 has been simplified to read as “Synthesis of the included studies identified prominent themes relating to the effects of immigration detention on health outcomes in ASR adults and children.”

Lines 553 impact on children:
While this is information from the studies, it is important to help the reader understand what you are trying to explain. Why are children not falling under the same category for psychological health and physical health?
Lines 712 – 719 talk about children. I am wondering why you are not putting this information together.

Authors’ Response: Thank you for your comment. We considered it important to identify a distinct subtheme for children, as the manifestation of mental disorders differs from that of adults, with these differences being particularly pronounced before adolescence. We have now prefaced this point in the manuscript on lines 851-880. 

“Of note, the latter subtheme was delineated to reflect the distinct presentation of mental disorders, the differing epidemiology of physical health issues, and the general recognition of children as a more vulnerable group.”

A separate section on Children and Families (4.1) lines 1531 – 1552 remains. 


Line 588: Protection is mentioned here, and it is the only time I have read anything in the paper on protection. Reading your themes, a way to bring back protection, legal, health, and social support would highlight the need for a multidisciplinary approach to this topic.
Authors’ Response: Thank you. We have now discussed the concept of protection earlier in the paper in the introduction line 187-201 in the context of ASR. 

We have also embedded this in section 4.5 Clinical Implications and Future research lines 1613- 1616:

“The results of this synthesis may prove beneficial for health professionals, legal practitioners, social workers and policymakers involved in immigration detention, as they can inform the development of future practices and interventions tailored to this population.” 


Flow chart: It should include all the databases searched and the grey literature.

Authors’ Response: Thank you for your comment. We have now amended this in the manuscript. 

Results: They do not mention anything linked to physical health, as explained under line 165, but the mental health of adults and children. I would have a similar remark for the conclusion, too.

Authors’ Response: We appreciate your feedback. We are aware that the review largely focuses on mental health, reflecting the paucity of studies addressing physical health in this population, particularly within the qualitative literature. 

We have now more clearly specified the limited number of studies (n=2) assessing physical health:

Lines 427- 429: “While all included studies addressed mental health, only two specifically reported on physical health issues [22, 76]”

Nonetheless, lines 849-891 and 1257-1259 of the Results section specifically address physical health issues. The limited availability of data on physical health, as well as the frequent conflation of physical and mental issues in the included studies, is further discussed in the Discussion (1564-1569; 1601-1603). Finally, the Conclusions emphasise the need for additional research on the physical health of detained ASR; lines 1623 – 1628:

“Asylum seekers and refugees, both during their period of detention and subsequently. While evidence on physical health outcomes is limited, the cumulative consequences of detention highlight the necessity for comprehensive support and healthcare services and associated clinicians and support staff that are tailored to the needs of individuals who have been detained.  The findings provide a synthesis of the current literature, illustrating the persistent psychological health implications linked to immigration detention. In contrast, observations regarding physical health have been infrequently documented throughout the studies.” 

6. PLOS authors have the option to publish the peer review history of their article (what does this mean?). If published, this will include your full peer review and any attached files.

Authors’ Response: We agree to the publication of the peer review history for this manuscript, with the understanding that reviewer anonymity will be maintained unless reviewers have themselves opted to be identified.

Do you want your identity to be public for this peer review? If you choose “no”, your identity will remain anonymous but your review may still be made public.

For information about this choice, including consent withdrawal, please see our Privacy Policy.
Reviewer #1: Yes: Norman Sitali SRN/MIH/MPM
